# DNABERT-S: pioneering species differentiation with species-aware DNA embeddings

**DOI:** 10.1093/bioinformatics/btaf188

**Published:** 2025-07-15

**Authors:** Zhihan Zhou, Weimin Wu, Harrison Ho, Jiayi Wang, Lizhen Shi, Ramana V Davuluri, Zhong Wang, Han Liu

**Affiliations:** Department of Computer Science, Northwestern University, Evanston, IL 60208, United States; Center for Foundation Models and Generative AI, Northwestern University, Evanston, IL 60208, United States; Department of Computer Science, Northwestern University, Evanston, IL 60208, United States; Center for Foundation Models and Generative AI, Northwestern University, Evanston, IL 60208, United States; School of Natural Science, University of California at Merced, Merced, CA 95343, United States; Department of Energy Joint Genome Institute, Lawrence Berkeley National Laboratory, Berkeley, CA 94720, United States; Department of Computer Science, Northwestern University, Evanston, IL 60208, United States; Center for Foundation Models and Generative AI, Northwestern University, Evanston, IL 60208, United States; Department of Statistics and Data Science, Northwestern University, Evanston, IL 60208, United States; Department of Biomedical Informatics, Stony Brook University, Stony Brook, NY 11794, United States; School of Natural Science, University of California at Merced, Merced, CA 95343, United States; Department of Energy Joint Genome Institute, Lawrence Berkeley National Laboratory, Berkeley, CA 94720, United States; Environmental Genomics and Systems Biology Division, Lawrence Berkeley National Laboratory, Berkeley, CA 94720, United States; Department of Computer Science, Northwestern University, Evanston, IL 60208, United States; Center for Foundation Models and Generative AI, Northwestern University, Evanston, IL 60208, United States; Department of Statistics and Data Science, Northwestern University, Evanston, IL 60208, United States

## Abstract

**Summary:**

We introduce DNABERT-S, a tailored genome model that develops species-aware embeddings to naturally cluster and segregate DNA sequences of different species in the embedding space. Differentiating species from genomic sequences (i.e. DNA and RNA) is vital yet challenging, since many real-world species remain uncharacterized, lacking known genomes for reference. Embedding-based methods are therefore used to differentiate species in an unsupervised manner. DNABERT-S builds upon a pre-trained genome foundation model named DNABERT-2. To encourage effective embeddings to error-prone long-read DNA sequences, we introduce Manifold Instance Mixup (MI-Mix), a contrastive objective that mixes the hidden representations of DNA sequences at randomly selected layers and trains the model to recognize and differentiate these mixed proportions at the output layer. We further enhance it with the proposed Curriculum Contrastive Learning (C^2^LR) strategy. Empirical results on 28 diverse datasets show DNABERT-S’s effectiveness, especially in realistic label-scarce scenarios. For example, it identifies twice more species from a mixture of unlabeled genomic sequences, doubles the Adjusted Rand Index (ARI) in species clustering, and outperforms the top baseline’s performance in 10-shot species classification with just a 2-shot training.

**Availability and implementation:**

Model, codes, and data are publically available at https://github.com/MAGICS-LAB/DNABERT_S.

## 1 Introduction

Accurate differentiation of species from genomic sequences is a critical task in biology and ecology, supporting efforts in biodiversity conservation, epidemiology, understanding evolutionary processes, and exploring the roles of microbiomes in health and disease. Traditional methods for species identification rely heavily on well-characterized reference genomes for comparative analysis. Thus, they are limited due to the vast and largely unexplored genetic diversity present in natural environments.

A prime example is metagenomics binning. Metagenomics binning (Kang *et al.* 2015, 2019, Nissen *et al.* 2021, Meyer *et al.* 2022, Lamurias *et al.* 2023) is a pivotal process in microbiome research, aiming to group DNA sequences by species from complex mixtures containing DNA from potentially thousands of distinct, often uncharacterized species. In this context, effective DNA embeddings that can accurately segregate and cluster DNA sequences are more suitable than the methods that rely on known reference genomes for comparison and alignment.

Despite the critical role of DNA embeddings in various scenarios, there is a notable deficiency in the development of effective methods. Current approaches to achieving DNA embeddings include: (i) Descriptive textual features (Kang *et al.* 2015, 2019, Nissen *et al.* 2021), (ii) Pre-trained Kmer embeddings (Ng 2017, Han *et al.* 2022, Ren *et al.* 2022), and (iii) Genome foundation models (Ji *et al.* 2021, Nguyen *et al.* 2023, Zhou *et al.* 2023). The first two methods, while straightforward, often fail to capture complex semantic relationships inherent in genomic data. Genome foundation models (Ji *et al.* 2021, Nguyen *et al.* 2023, Zhou *et al.* 2023), despite their successes in various genomic tasks through model fine-tuning, generally fail to develop embeddings that can discriminate certain properties like species. This is largely due to a mismatch between their pre-training objectives (Devlin *et al.* 2018, Radford *et al.* 2019) and the specific application scenarios. Our empirical analysis, as detailed in Table 1, reveals that in many scenarios, existing genome foundation models even underperform simple textual features.

**Table 1.** Models’ performance on K-Means clustering measured by Adjusted Rand Index (ARI).^a^

Model	Synthetic		Marine					Plant					Ave.
	0	1	0	1	2	3	4	0	1	2	3	4	
**TNF**	38.75	37.76	25.65	25.31	26.05	20.67	23.47	25.80	24.23	24.81	22.72	22.39	26.47
**TNF-K**	36.26	35.66	25.99	25.00	26.27	21.15	23.27	25.60	25.58	26.45	22.59	21.76	26.30
**TNF-VAE**	25.94	24.60	16.28	16.52	16.27	12.92	15.02	18.40	16.51	17.53	14.08	14.38	17.37
**DNA2Vec**	24.68	23.34	16.07	15.99	16.18	12.62	14.51	20.13	19.77	20.25	17.24	16.37	18.10
**HyenaDNA**	20.04	18.99	16.54	16.64	16.47	13.35	14.85	24.06	25.33	26.18	21.01	21.16	19.55
**NT-v2**	8.69	9.63	4.92	4.74	5.02	3.68	4.31	7.00	6.32	6.37	5.54	5.42	5.97
**DNABERT-2**	15.73	16.74	13.24	13.53	12.99	10.41	11.87	15.70	16.28	16.32	13.99	13.66	14.21
**DNA-Dropout**	16.64	16.08	11.89	11.77	11.89	9.85	10.31	16.18	15.41	16.95	13.53	13.85	13.70
**DNA-Double**	35.11	34.14	27.05	27.23	26.56	21.47	24.39	22.35	21.35	23.03	19.44	19.06	25.10
**DNA-Mutate**	16.55	16.24	11.40	11.53	11.34	9.03	10.02	14.27	14.13	16.22	12.01	11.68	12.87
**DNABERT-S**	**68.21**	**66.33**	**53.98**	**52.56**	**51.99**	**46.39**	**50.49**	**51.43**	**51.56**	**51.11**	**50.44**	**51.15**	**53.80**

a DNABERT-S doubles the ARI of the strongest baseline on average. Bold text indicates the one with the highest score.

**Figure 1.**
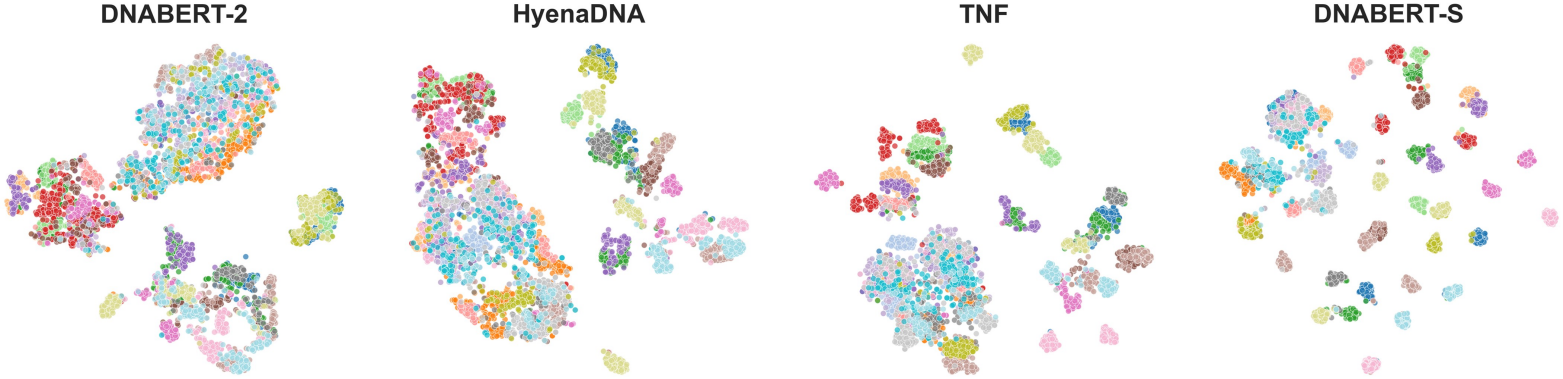
TSNE visualization of the DNA embeddings generated by different methods on a CAMI2 (Meyer *et al.* 2022) dataset with 50 different species. Each point represents an individual DNA sequence, with the color coding indicating the species affiliation. Notably, DNABERT-S demonstrates a pronounced ability to cluster and segregate different species within the embedding space.

In this work, we introduce DNABERT-S, a specialized genome model that harnesses the capabilities of genome foundation models to generate species-aware DNA embeddings. As depicted in Fig. 1, DNABERT-S distinguishes itself from other methods by its ability to effectively cluster and separate different species within the embedding space. This enhanced performance stems from the proposed Manifold Instance Mixup (MI-Mix) loss and Curriculum Contrastive Learning (C^2^LR) strategy. Contrastive learning enables the model to discern between similar and dissimilar DNA sequences, and curriculum learning incrementally presents more challenging training samples, fostering better learning and generalization. The training of DNABERT-S includes two phases. In the first phase, we adopt a Weighted SimCLR (Chen *et al.* 2020, Zhou *et al.* 2022) training objective to encourage the model to group DNA sequences from the same species and separate DNA sequences from distinct species. In the second phase, we introduce Manifold Instance Mixup (MI-Mix), which mixes anchor instances at a randomly selected layer to create more challenging anchors for contrastive training.

To rigorously evaluate the models, we assemble a comprehensive benchmark that includes thousands of species, capturing the diversity of natural microbial communities. This benchmark incorporates complex samples from CAMI2 (Meyer *et al.* 2022), a leading metagenomics binning benchmark, and extensive reference genomes from Genbank (Benson *et al.* 2013). Thus, it includes both natural yet error-prone long-read sequences and well-curated yet potentially biased reference genomes.

We evaluate the embedding quality from entirely unsupervised problems to classification tasks with abundant labels. Experimental results indicate the effective performance of DNABERT-S. Compared to the strongest existing method, DNABERT-S doubles its performance in the clustering task and achieves better performance with only 20% of labeled data in the classification task (e.g. 2-shot versus 10-shot). Notably, in metagenomics binning, DNABERT-S is able to recover over 40% and 80% of species with an F1 score of over 0.5, respectively, from synthetics and more realistic datasets, which is also one time more than the strongest baseline. We also show that a simple K-Nearest-Neighbors species classifier, which is trained on DNABERT-S embeddings of a small portion of each target genome, can slightly outperform a well-established traditional method MMseqs2 (Steinegger and Söding 2017) that relies on the entire reference genomes of target species for classification. Besides, with DNABERT-S embeddings as input and without using alignment or abundance information, we achieve comparable or slightly better performance in metagenomics binning as SemiBin2 (Pan *et al.* 2023), an advanced and complete metagenomics binning tool.

Our contribution can be summarized as follows: (i) We demonstrate the effectiveness of genome foundation models in learning DNA embeddings, opening new avenues for various genomics research problems; (ii) We introduce DNABERT-S, a model that develops distinctly better embeddings for species differentiation; (iii) We propose the Curriculum Contrastive Learning (C^2^LR) strategy with the Manifold Instance Mixup (MI-Mix) loss; (iv) We publish a large-scale evaluation benchmark.

## 2 Background

This study aims to build a species-aware DNA embedding model that maps each DNA sequence as a fixed-size numerical vector in an embedding space, where sequences from distinct species are naturally clustered and segregated. A DNA sequence is essentially a sentence composed of four unique characters: A, T, C, and G.

Existing works highly rely on descriptive textual features (Kang *et al.* 2015, 2019, Nissen *et al.* 2021) and pre-trained K-mer embeddings (Ng 2017, Han *et al.* 2022, Ren *et al.* 2022) to compute DNA embeddings. A representative descriptive textual feature is Tetra-Nucleotide Frequency (TNF), a 256D vector where each position represents the frequency of each unique 4-mer (e.g. TTCA, AACG) in the input DNA sequence. Despite its simplicity and effectiveness, this method is limited since it singly relies on the 4-mer frequency and is not trainable to better fit downstream applications. Besides, our empirical analysis also suggests that a naive trainable model based on TNF, such as a Variational AutoEncoder (Diederik *et al.* 2013) with TNF as input, results in worse embeddings compared to TNF. With the success of Word2Vec (Mikolov *et al.* 2013), pre-trained Kmer embeddings have gained popularity in computing DNA embeddings for various applications (Ng 2017, Han *et al.* 2022, Ren *et al.* 2022). However, the emergence of deep learning advancements such as ELMo and BERT (Devlin *et al.* 2018, Peters *et al.* 2018) highlights the limitations of static word embeddings compared to contextual embeddings produced by foundation models.

Recently, genome foundation models such as DNABERT-2 and HyenaDNA have demonstrated their effectiveness in genome analysis (Ji *et al.* 2021, Dalla-Torre *et al.* 2023, Nguyen *et al.* 2023, Zhou *et al.* 2023). However, these models do not naturally develop discriminative embeddings, largely due to the discrepancy between their language-modeling training objectives and the goal of segregating sequences in the embedding space (Li *et al.* 2020). To leverage the power and potential of genome foundation models, we tailor a contrastive learning method (Reimers and Gurevych 2019, Chen *et al.* 2020, Lee *et al.* 2020, Gao *et al.* 2021) for DNA embedding learning by introducing the curriculum contrastive learning (C^2^LR) strategy with the Manifold Instance Mixup (MI-Mix) training objective.

## 3 Model

The proposed Curriculum Contrastive Learning ($C^{2}$LR) splits the training process into two phases, gradually creating more challenging anchors. In phase I, we apply an effective contrastive learning method named Weighted SimCLR based on SimCLR and Hard-Negative sampling strategy (Section 3.1). In phase II, we propose the Manifold Instance Mixup method which creates more challenging anchors by mixing intermediate hidden states of inputs in a randomly selected hidden layer of the model (Section 3.2). Implementation details of DNABERT-S are presented in Section 3.3.

*Notation:* Let $x_{i}$ be an input sample. Given a batch ${\left\{(x_{i},x_{i^{+}})\right\}}_{i=1}^{B}$, where *B* is the batch size and $\left(x_{i},x_{i^{+}}\right)$ represents a pair of similar samples (a.k.a., positive pair). In our setting, a positive pair $\left(x_{i},x_{i^{+}}\right)$ represents two nonoverlapping DNA sequences from the same genome. Let f(·) define the embedding model, which takes $x_{i}$ as input and computes fixed-size embedding $f(x_{i})$.

### 3.1 Weighted SimCLR

SimCLR (Chen *et al.* 2020) is a simple and effective framework for contrastive learning. For an anchor $x_{i}$ in batch ${\left\{(x_{i},x_{i^{+}})\right\}}_{i=1}^{B}$, SimCLR treats all the other 2B−2 samples in the same batch as negative samples. It encourages the model to increase the anchor’s similarity with its positive sample $x_{i^{+}}$ and reduces its similarity with the negative samples. It treats all negative samples equally. However, recent works (Zhang *et al.* 2021) have suggested that hard negatives that are closer to the anchor in the representation space offer more informative learning contrasts. Therefore, Weighted SimCLR (Zhang *et al.* 2021) gives higher weights to negative samples that are closer to the anchor. To align with subsequent sections, we introduce the virtual labels. The label for $\left(x_{i},x_{i^{+}}\right)$ is $v_{i}\in {\left\{0,1\right\}}^{B}$, where $v_{i,i}=1$ indicates positive samples, and $v_{i,j\neq i}=0$ indicates negative samples. The Weighted SimCLR loss for $x_{i}$ is defined as:


(1) $$\ell (f(x_{i}),v_{i})=-\sum_{n=1}^{B}v_{i,n}\;\text{log}\;\frac{\;\text{exp}\;\left(s\left(f(x_{i}),f(x_{n^{+}})\right)/\tau \right)}{\sum_{j\neq i}\alpha_{ij}\;\text{exp}\;\left(s\left(f(x_{i}),f(x_{j})\right)/\tau \right)},$$

where τ denotes the temperature and s(·,·) denotes the cosine similarity between two inputs. Weights $\alpha_{ij}$ denotes the relative importance of $x_{j}$ for optimizing the contrastive loss of anchor $x_{i}$ among all the 2B−2 negative samples. A negative sample that is closer to the anchor receives a higher weight. We set $\alpha_{ii^{+}}=1$ and compute $\alpha_{ij}$ as:


$$\begin{array}{cc} & \alpha_{ij}=\frac{\;\text{exp}\;\left(s\left(f(x_{i}),f(x_{j})\right)/\tau \right)}{\frac{1}{2B-2}\sum_{k\neq i,i^{+}}\;\text{exp}\;\left(s\left(f(x_{i}),f(x_{k})\right)/\tau \right)}. \end{array}$$

For each positive pair $\left(x_{i},x_{i^{+}}\right)$, Weighted SimCLR, respectively, takes $x_{i}$ and $x_{i^{+}}$ as the contrastive anchors to calculate the contrastive loss. It defines the loss $\ell (f(x_{i^{+}}),v_{i})$ for $x_{i^{+}}$ by exchanging the roles of instances ${\left\{x_{i}\right\}}_{i=1}^{B}$ and ${\left\{x_{i^{+}}\right\}}_{i=1}^{B}$ in Equation (1), respectively. Therefore, the Weighted SimCLR loss on the entire batch is defined as:


(2) $$L=\frac{1}{2B}\sum_{i=1}^{B}\left(\ell (f(x_{i}\right),v_{i})+\ell (f(x_{i^{+}}),v_{i})).$$

### 3.2 Curriculum contrastive learning

In this part, we introduce our curriculum contrastive learning ($C^{2}$LR) method. Curriculum learning is an effective training method that first presents easy training batches and then progresses to more challenging ones (Hacohen and Weinshall 2019). Recent studies have successfully applied this technique to both positive pairs (Ye *et al.* 2021, Roy and Etemad 2023) and negative pairs (Chu *et al.* 2021) in contrastive learning. We take this approach a step further by applying it to contrastive anchors, effectively using it for both types of pairs at the same time.

**Figure 2.**
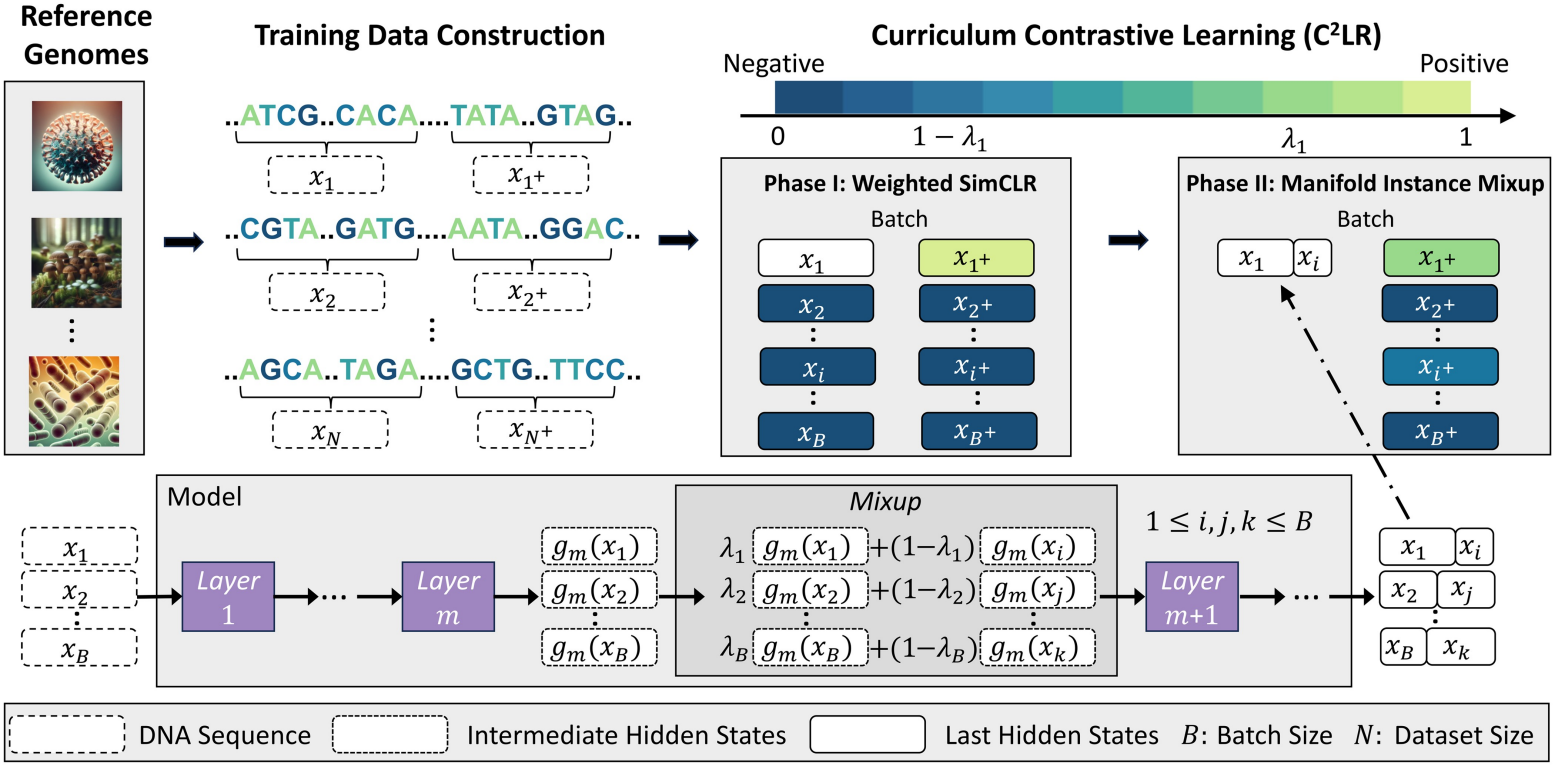
Overview of DNABERT-S’s training process. We construct training data from massive reference genomes and train DNABERT-S with the proposed Curriculum Contrastive Learning (C^2^LR) strategy that progressively provides more challenging contrastive anchors to the model in two different phases. We propose the Manifold Instance Mixup (MI-Mix) objective that mixes the intermediate hidden states of different inputs to construct more challenging contrastive anchor.

As shown in Fig. 2, our $C^{2}$LR method includes two training phases, with anchors becoming progressively more challenging. In phase I, we use the Weighted SimCLR introduced in Section 3.1. In phase II, we propose the Manifold Instance Mixup (MI-Mix) method to mix up anchor instances in a random hidden layer, motivated by the instance mixup (i-Mix) method (Lee *et al.* 2020).

The i-Mix method mixes anchors at the input layer to create more challenging positive and negative pairs. It only uses the samples from ${\left\{x_{i}\right\}}_{i=1}^{B}$ as anchors and only considers the positive and negative samples from ${\left\{x_{i^{+}}\right\}}_{i=1}^{B}$. Otherwise, it nearly doubles the memory or training time compared to the Weighted SimCLR method in Section 3.1 (see Supplementary Appendix S10 for details). To perform mixup within the anchor space, i-Mix first shuffles ${\left\{(x_{i},v_{i})\right\}}_{i=1}^{B}$ to generate ${\left\{({\hat{x}}_{i},{\hat{v}}_{i})\right\}}_{i=1}^{B}$, i.e. a random permutation of ${\left\{(x_{i},v_{i})\right\}}_{i=1}^{B}$. Then for each anchor $\left(x_{i},v_{i}\right)$, i-Mix mixes it with $\left({\hat{x}}_{i},{\hat{v}}_{i}\right)$ through weighted sum. The mixing weight $\lambda_{i}$ is drawn from Beta(α,α), where α is a hyperparameter.

Despite i-Mix’s effectiveness on continuous data such as images and speeches, directly mixing DNA sequences may avoid biological plausibility. Thus, we proposed to instead mix hidden representations of DNA sequences at a deeper layer, which essentially combines more abstract, higher-level features of the sequences. We call it Manifold Instance Mixup, inspired by Manifold mixup (Verma *et al.* 2019). Concretely, we denote the model f(·) as $f(x)=f_{m}(g_{m}(x))$. Here, $g_{m}(\cdot )$ maps input data to the intermediate hidden states at layer *m*, and $f_{m}(\cdot )$ maps these intermediate hidden states to the output f(x).

The Manifold Instance Mixup includes four steps. First, we uniformly select a random layer *m* from a set of eligible layers S in the model, like one of the encoder layers in DNABERT-S. Second, for a batch of anchors ${\left\{(x_{i},v_{i})\right\}}_{i=1}^{B}$, we process them up to layer *m*, resulting in a batch of intermediate hidden states ${\left\{(g_{m}(x_{i}),v_{i})\right\}}_{i=1}^{B}$. Third, we shuffle ${\left\{(g_{m}(x_{i}),v_{i})\right\}}_{i=1}^{B}$ to get ${\left\{(g_{m}({\hat{x}}_{i}),{\hat{v}}_{i})\right\}}_{i=1}^{B}$ and mix them up. This produces the mixed hidden states $h_{i}^{m}$ for each $\left(g_{m}(x_{i}),v_{i}\right)$, where $h_{i}^{m}=\lambda_{i}g_{m}(x_{i})+(1-\lambda_{i})g_{m}({\hat{x}}_{i})$, and $v_{i}^{\mathrm{mix}}=\lambda_{i}v_{i}+(1-\lambda_{i}){\hat{v}}_{i}$. Fourth, we feed ${\left\{h_{i}^{m}\right\}}_{i=1}^{B}$ through the remaining layers to get the last hidden states ${\left\{f_{m}(h_{i}^{m})\right\}}_{i=1}^{B}$. Loss on *i*-th anchor $\left(x_{i},v_{i}\right)$ is defined as:


(3) $$\begin{array}{c} \;\hat{\ell }(f_{m}(h_{i}^{m}),v_{i}^{\mathrm{mix}})\\ =-\sum_{n=1}^{B}v_{i,n}^{\mathrm{mix}}\;\text{log}\;\frac{\;\text{exp}\;\left(s\left(f_{m}(h_{i}^{m}),f(x_{n^{+}})\right)/\tau \right)}{\sum_{j=1}^{B}\alpha_{ij^{+}}\cdot \;\text{exp}\;\left(s\left(f_{m}(h_{i}^{m}),f(x_{j^{+}})\right)/\tau \right)}, \end{array}$$

where weights $\alpha_{ii^{+}}=1$ and $\alpha_{ij^{+}}$ is computed as:


$$\begin{array}{cc} & \alpha_{ij^{+}}=\frac{\;\text{exp}\;\left(s\left(f_{m}(h_{i}^{m}),f(x_{j^{+}})\right)/\tau \right)}{\frac{1}{B-1}\sum_{k=1,k\neq i}^{B}\;\text{exp}\;\left(s\left(f_{m}(h_{i}^{m}),f(x_{k^{+}})\right)/\tau \right)}. \end{array}$$

The Manifold Instance Mixup loss is defined as follows:


(4) $$\hat{L}=\frac{1}{B}\sum_{i=1}^{B}\hat{\ell }(f_{m}(h_{i}^{m}),v_{i}^{\mathrm{mix}}).$$

### 3.3 Implementation

In the $C^{2}$LR method, we set temperature τ as 0.05 and hyperparameter α as 1.0. We train the model for one epoch in phase I using loss Equation (2) and for two epochs in phase II using loss Equation (4). We use mean pooling of the last hidden states of all the tokens as the DNA embedding. We employ the Adam optimizer (Diederik *et al.* 2014), with a learning rate of 3e−6 and batch size of 48. We save the model every 10 000 training steps and select the best one based on the validation loss in the validation dataset. We use the pre-trained DNABERT-2 (Zhou *et al.* 2023) as the starting point of contrastive training. We also conduct parallel experiments with HyenaDNA (Nguyen *et al.* 2023). In Section 5.7, we show that DNABERT-2 outperforms HyenaDNA after the same contrastive training. The training of DNABERT-S takes approximately 48 h on 8 NVIDIA A100 80GB GPUs.

## 4 Data

In this section, we introduce the dataset we used for DNABERT-S training and evaluation.

### 4.1 Training

Each training sample of DNABERT-S is a pair of nonoverlapping DNA sequences extracted from the same species. We focus on microbe species since the genetic diversity within this group provides a rich substrate for examining the nuances of species differentiation. The dataset is constructed with the reference genomes from GenBank (Benson *et al.* 2013). We obtained 47 923 pairs from 17 636 viral genomes, 1 million pairs from 5011 fungi genomes, and 1 million pairs from 6402 bacteria genomes. We randomly selected 2 million pairs from the entire 2 047 923 pairs of DNA sequences to construct the training data. The rest pairs are treated as validation data. All the DNA sequences are 10 000 bp in length.

### 4.2 Evaluation

Our evaluation spans 14 long-read datasets from the Critical Assessment of Metagenome Interpretation (CAMI) II (Meyer *et al.* 2022) challenge benchmark and nine synthetic datasets from reference genomes. CAMI2 is one of the most comprehensive and rigorous benchmarks for metagenomics research. The datasets in CAMI2 are designed to mimic realistic microbiome environments and include a vast array of both new and known genomes, as well as plasmids and viruses. It aligns our study with real-world ecological and biological scenarios, providing a robust and contextually relevant evaluation for the DNA embedding models. We utilize seven datasets of long-read contigs, respectively, from the Marine and Plant-associated environments, where each dataset consists of 150k–200k DNA sequences belonging to about 100–750 different species sampled from 1680 microbial genomes and 599 circular elements. We also create nine Synthetic datasets by randomly extracting DNA sequences from fungi and viral reference genomes that *do not overlap* with our training data. Supplementary Table S5 in Supplementary Appendix S8 shows the statistics of the datasets we used for evaluation.

## 5 Experiments

We evaluate the model in a series of tasks, including: (i) metagenomics binning that identifies species from a mixture of sequences from an unknown number of species; (ii) species clustering given the number of species; (iii) species classification with a few labeled samples; and (iv) long-read classification given reference genomes. For the metagenomics binning problem, to mimic real-world scenarios, we do not balance the data. Instead, following Metabat (Kang *et al.* 2015), we only keep DNA sequences longer than 2500 bp and filter out species with fewer than 10 sequences. Furthermore, we validate the absence of data leakage issue in our evaluation datasets. Please refer to Supplementary Appendix S12 for details.

In this section, we present experimental design and empirical results. We introduce baselines in Section 5.1 and, respectively, present the results of clustering in Section 5.2, metagenomics binning in Section 5.3, and classification in Section 5.4. In Section 5.5, we present ablation studies on C^2^LR and the proposed Manifold Instance Mixup training objective. Comparison with an alignment-based method for species classification is presented in Supplementary Appendix S9.4. We also provide empirical analysis on results with error bars (Supplementary Appendix S9.3), scenarios with abundant training data (Supplementary Appendix S9.4), results on nonmicrobe species (Supplementary Appendix S9.5), different input lengths (Supplementary Appendix S9.6), reduced feature dimensions (Supplementary Appendix S9.7), various other types of tasks (e.g. genomics function prediction) and varying backbone models (Supplementary Appendix S9.8). For all tasks involving randomness, we perform five independent runs with different random seeds for each model and report the averaged results.

### 5.1 Baselines

We compare DNABERT-S with other DNA embedding models to examine its effectiveness. We consider four lines of work. *TNF, TNF-K*, and *TNF-VAE* are the most widely used DNA embedding methods in metagenomics binning tools (Kang *et al.* 2015, 2019, Nissen *et al.* 2021). *TNF* represents Tetra-Nucleotide Frequency, which uses the appearance frequency of each unique 4-mer ($4^{4}=256$ in total) in a DNA sequence as its embedding. *TNF-K* (Nissen *et al.* 2021) reduces TNF to 103D with a linear kernel, which utilizes DNA characteristics to reduce the correlations among different dimensions of the original TNF feature. *TNF-VAE* trains a Variational Autoencoder (Diederik *et al.* 2013) using TNF as input to extract features. *DNA2Vec* (Ng 2017) learns pre-trained K-mer embedding. We set K=4 to make it directly comparable with TNF and use the average of the 4-mer embeddings as the DNA embedding. *DNABERT-2* (Zhou *et al.* 2023), *HyenaDNA* (Nguyen *et al.* 2023), and *NT-v2 (Nucleotide Transformer-v2)* (Dalla-Torre *et al.* 2023) are representative genome foundation models. We use the average of the last hidden states as the DNA embedding. For evaluations, we utilized the respective pre-trained models from Huggingface ModelHub, specifically *zhihan1996/DNABERT-2-117M*, *LongSafari/hyenadna-medium-450k-seqlen-hf*, and *InstaDeepAI/nucleotide-transformer-v2-100m-multi-species*. *DNA-Mutate, DNA-Dropout*, and *DNA-Double* are variants of DNABERT-S, with the same hyperparameters but different positive pair construction strategies in contrastive training. *DNA-Mutate* views the same DNA sequence before and after random mutation (i.e. swap and delete 5% of nucleotides) as a positive pair. *DNA-Dropout* passes the same DNA sequence through the embedding model (with a dropout rate 0.1) twice and views the two distinct embeddings as a positive pair. *DNA-Double* views a DNA sequence and its reverse complementary (e.g. AATTC versus TTAAG) as a positive pair. We also compare the proposed curriculum contrastive learning framework and MI-Mix training objective with well-established contrastive learning methods, including *Weight SimCLR* (Zhang *et al.* 2021), *i-Mix* (Lee *et al.* 2020), and *Supervised Contrastive Learning (SupCon)* (Khosla *et al.* 2021). Results on different training objectives are presented in Section 5.5.

### 5.2 Clustering

In this task, we evaluate the embedding quality by how well a standard clustering algorithm can distinguish and cluster different species based on the embedding. To reduce the effects of other factors, we assume the number of species is known in this task. Although we are unlikely to know the number of species in a real-world dataset, this setup allows us to fairly compare different embedding models. The metagenomics binning experiments discussed in Section 5.3 represent a more realistic metagenomics problem. For each dataset, we compute the embedding of each DNA sequence and perform K-means clustering by setting the num_clusters as the number of species that exist in this dataset. We employ the Adjusted Rand Index (ARI) as the evaluation metric. ARI is a measure of the similarity between two data clusterings, adjusted for chance, providing a normalized index that ranges from −1 to 1; the higher, the better.

Table 1 shows the models’ performance on clustering. As shown in the table, DNABERT-S consistently achieves the best performance on all the datasets and doubles the performance of the strongest existing method on average. Among all the baselines, TNF and its variant TNF-K achieve the best performance, explaining their wide usage in metagenomics binning. Yet, TNF’s performance is heavily limited since it is not learnable. TNF-VAE represents a naive algorithm that enables learning with TNF, yet it leads to big performance degradation, potentially resulting from the large gap between its training objective and the specific downstream application. Similarly, pre-trained Kmer embeddings from DNA2Vec also fail to effectively cluster different species.

Existing genome foundation models training with language modeling objectives, such as HyenaDNA and DNABERT-2, despite their good performance on labeled datasets, also fail to generate representative embedding without fine-tuning. The phenomenon that pre-trained foundation models underperform descriptive textual features in generating embedding for clustering and retrieval is also observed in the field of natural language processing (Reimers and Gurevych 2019).

Furthermore, by comparing the DNA-Dropout and DNA-Mutate with DNABERT-2, we found that those popular unsupervised positive pair methods used in contrastive learning in NLP, such as sentence swap/deletion and dropout, do not benefit DNA embedding learning. The DNA-Double, which utilizes the unique double-strain characteristics of DNA sequences, empowers DNABERT-2 to achieve a similar level of performance as TNF. Comparison between DNABERT-S and these variants indicates the importance of appropriate training data construction.

### 5.3 Metagenomics binning

Metagenomics binning is a crucial process in microbial ecology, involving the categorization of DNA sequences into groups that represent individual species. State-of-the-art metagenomics binning method (Kang *et al.* 2015, 2019, Nissen *et al.* 2021) always formulate this problem as a clustering problem with an unknown number of clusters based on the feature of each DNA sequence. The DNA sequence feature is computed by combining sequence-based DNA embedding with various other features and the clustering algorithms are often complicated and strongly correlated with the features they utilize. In our evaluation, to create a fair environment for DNA embedding benchmarking, instead of relying on any existing tool, we implement the modified K-medoid clustering algorithm proposed in Metabat (Kang *et al.* 2015) for metagenomics binning due to its simplicity and effectiveness. Algorithm 1 (Supplementary Section S7 in Supplementary Material) describes the metagenomics binning algorithm. Following previous works (Kang *et al.* 2015, 2019), we formulate this problem as identifying nonoverlapping clusters of DNA sequences from the entire dataset, where each cluster of sequence is considered as an identified species. We iteratively identify the densest point in the embedding space and take all the sequences that are close (determined by a learned threshold) to it as the group of the sequences that belong to the same species. The embeddings of the taken sequences are removed from the embedding space. The iteration ends as there are no regions that contain enough number sequences within the threshold. This algorithm, evaluates how well the embedding method clusters and aggregates different species within the embedding space. We then compare the predicted clusters with the true labels to count the number of species that have been successfully identified. A species is considered to be successfully identified if the F1 score of this species is over 0.5. We compare different models by the number of species they identify with different levels of F1 scores (e.g. 0.5−0.6, 0.8−0.9). We only use the DNA embeddings as the feature of each DNA sequence.

**Figure 3.**
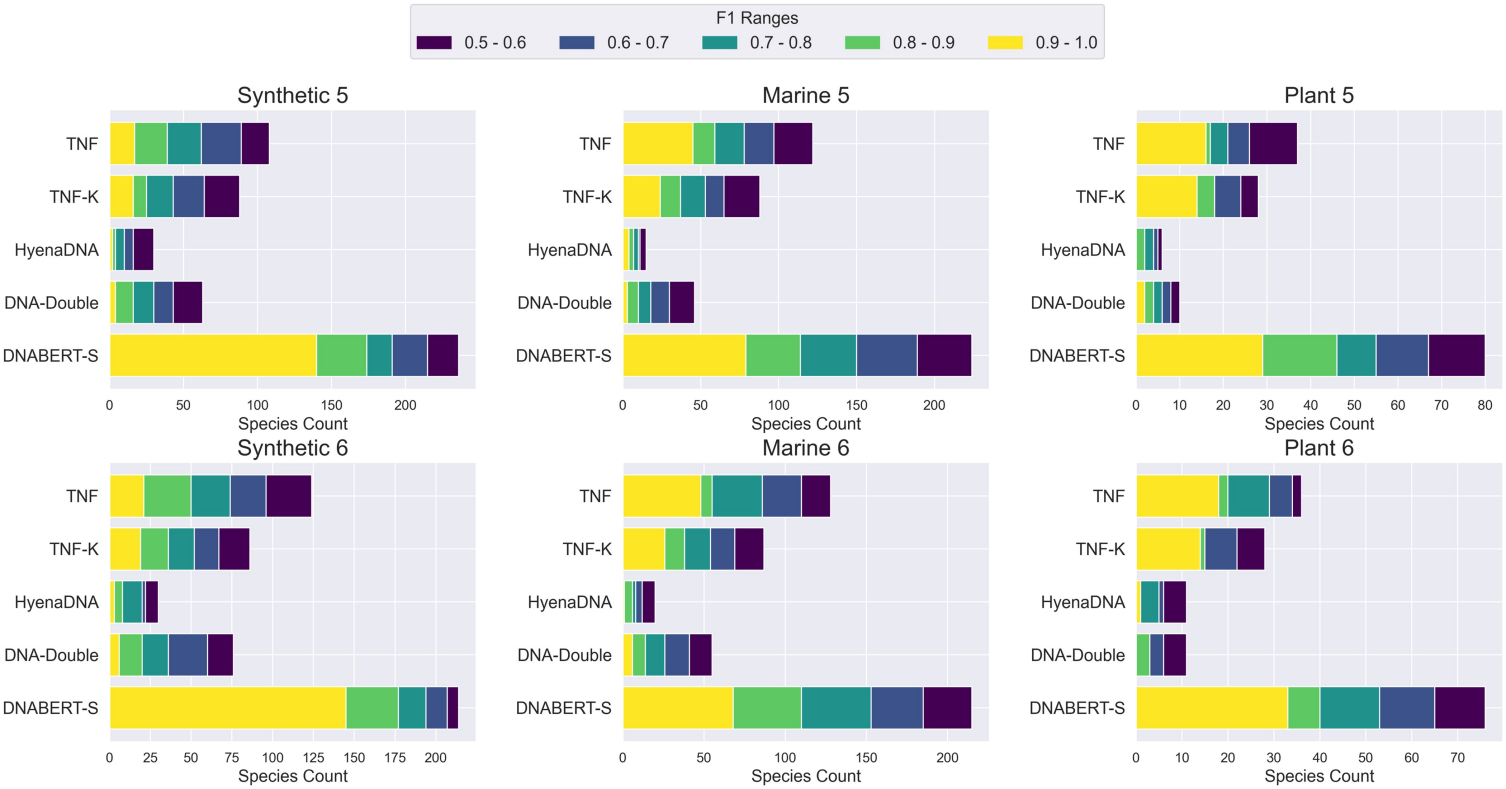
Metagenomics binning results. The bin size represents the number of unique species identified by each model, and different colors represent the F1 score of the identified species. With high F1 scores, DNABERT-S identifies more species than the baselines.

Figure 3 shows the models’ performance on 6 metagenomics binning datasets. As shown in the figure, similar to our observation in clustering, DNABERT-S identifies twice the number of species with an F1 score of over 0.5 compared to the strongest baseline, showing its great capability in tackling important real-world biology challenges. Notably, DNABERT-S identifies a large number of species with an F1 score over 0.9. indicating its capability to accurately segregate different species in the embedding space, aligning with our observation in Fig. 1. In the Synthetic datasets, where the sequences are error-less (extracted from reference genome) and the number of sequences in each species is more balanced, DNABERT-S recovers over 80% of the species with an F1 score of over 0.5 purely based on the DNA sequences themselves. In more realistic datasets such as Marine and Plant, where noise (e.g. error from sequences) exists in DNA sequence and species size is highly imbalanced, DNABERT-S is still able to recover 40% of the species with an F1 score of over 0.5.

### 5.4 Classification

In this task, we evaluate the embedding quality by how well a simple model can classify different species based on a few labeled embeddings. We conduct experiments with a linear regression model and a nonlinear multi-layer perceptron (MLP) to examine the embeddings’ linear and nonlinear descriptiveness. We present results on linear classification in this section. The results in nonlinear settings are consistent with those in linear ones. Due to space limits, we present them in Supplementary Tables S6 and S7 in Supplementary Appendix S9.2. As shown in Supplementary Table S5, all the datasets we use for classification consist of 100 DNA sequence for each species. We first compute the embedding of each DNA sequence with each model. In each evaluation run, we independently select 80 embeddings from each species to form the test set. For the rest DNA sequences, we, respectively, sample 1, 2, 5, 10, and 20 embeddings from each species to form the training set. A Logistic Regression model is trained on the training set and evaluated on the test set. We use the macro F1 score as the evaluation metric.

**Figure 4.**
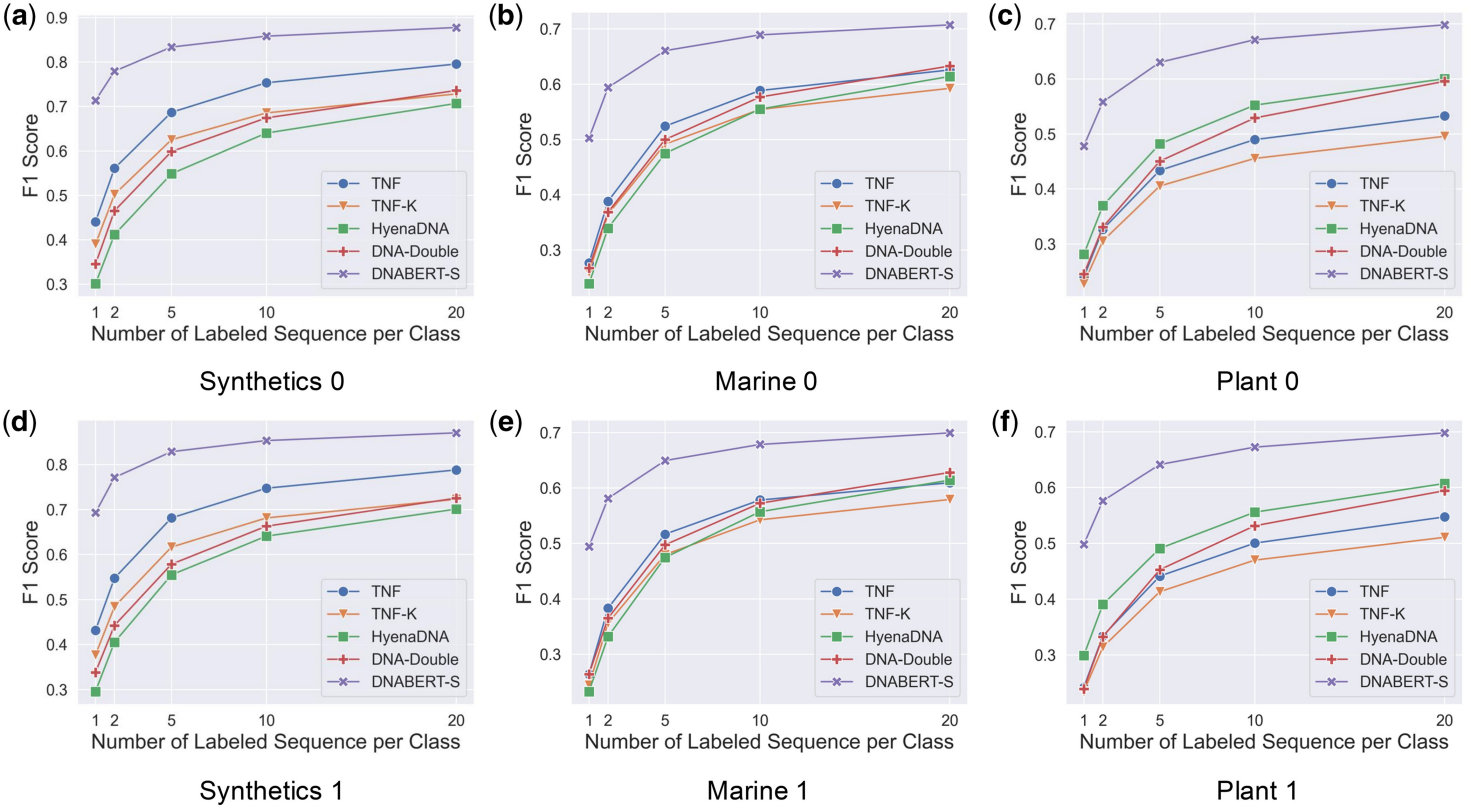
Model’s performance of species classification with varying numbers of training samples on six datasets. Results on other six datasets are consistent and are presented in Supplementary Fig. S6 (Supplementary Appendix S9.1 in the Supplementary Material).

Figure 4 shows the models’ performance on 6 datasets. The results for the remaining 6 datasets are consistent and are presented in Supplementary Appendix S9.1. As shown in the figure, DNABERT-S consistently achieves the best performance. DNABERT-S achieves better performance than the strongest baseline with only 20% of training data. For example, with only 2 training samples per category, DNABERT-S achieves higher F1 scores than the strongest baseline with 10 training samples. With the same amount of training samples, DNABERT-S outperforms the baselines by a large gap. Notably, in the Synthetic datasets, where none of the species are seen during the contrastive training, a linear model trained with DNABERT-S embeddings achieves an F1 score of over 0.8 in 200 classes classification with only 5 labeled samples in each species, showing DNABERT-S’s capability in generalizing well on unseen data. To further validate the generalizability of DNABERT-S on more distinct datasets, we compile three datasets, including genomes from invertebrate, protozoa, and mammalian species, that are largely different from the training species (microbial genomes) of DNABERT-S. As illustrated in Supplementary Section S9.5, DNABERT-S also achieves good performance in these datasets.

### 5.5 Ablation study

In this section, we present our ablation studies on DNABERT-S. We perform the ablation study on CAMI2 datasets with both clustering and classification. To validate the effectiveness of curriculum learning, we compare DNABERT-S with three of its variants, each of which is trained purely with the Weight SimCLR (Zhang *et al.* 2021), i-Mix (Lee *et al.* 2020), SupCon (Khosla *et al.* 2021), and our proposed Manifold Instance Mixup (MI-Mix) loss. To examine the effectiveness of MI-Mix, we also compare it with a variant trained with the curriculum contrastive method that replaces MI-Mix with i-Mix in the second phase. All the variants are trained with the same data and hyperparameters.

As shown in Table 2, our curriculum learning strategy that combines Weighted SimCLR and MI-Mix achieves the best performance. Our method outperforms both variants that are trained purely with Weighted SimCLR and MI-Mix loss, showing the effectiveness of our proposed curriculum contrastive learning strategy. Moreover, the comparison among the four variants that are trained with a single loss function, including Weight SimCLR, i-Mix, and SupCon, indicates the effectiveness of MI-Mix in learning DNA embeddings.

**Table 2.** Ablation study on the Curriculum Contrastive Learning (C^2^LR) and Manifold Instance Mixup (MI-Mix).^a^

Training objective	Clustering	Classification
W. SimCLR + MI-Mix	51.11	60.88
W. SimCLR + i-Mix	−3.46	−3.56
only W. SimCLR	−1.13	−1.17
only MI-Mix	−0.66	−0.42
only i-Mix	−5.25	−4.76
only SupCon	−6.75	−3.83

a Performance difference to W. SimCLR + MI-Mix.

### 5.6 Compare with complete metagenomics binning tool

Despite metagenomics binning being a main motivation of this work, DNABERT-S is not a complete metagenomics binning tool, making direct comparisons inappropriate. Modern metagenomics binning methods [e.g. MetaBat2 (Kang *et al.* 2019), SemiBin2 (Pan *et al.* 2023), and COMEBin (Wang *et al.* 2024)] typically follow a five-step pipeline utilizing three data types: (i) Generate DNA embeddings from sequences (*contigs*); (ii) Extract abundance information (*alignment files*); (iii) Combine DNA embeddings and abundance information to generate final embeddings; (iv) Perform clustering; (v) Refine results using external features (*e.g. length, single-copy gene markers*).

DNABERT-S specifically targets step 1 rather than end-to-end metagenomics binning. To evaluate its effectiveness, we implemented step 4 using the straightforward approach detailed in Algorithm 1 (Supplementary Section S7 in Supplementary Material), comparing DNABERT-S against other methods for step 1 (sequence embedding) only. We deliberately omitted steps 2, 3, and 5 to isolate and assess the impact of DNA embeddings alone. Despite our approach using much less available information (sequences only, without alignments or external features) and employing a simplified clustering algorithm, compared to complete binning methods like SemiBin2, we provide some comparison results with SemiBin2 on five CAMI2 samples to illustrate its effectiveness. Since SemiBin2 was mainly designed for short-read contigs, while our model is trained and evaluated primarily on long-read contigs, we collect five extra samples from the CAMI2 plant-associated dataset that contain short-read contigs. We apply the same evaluation criterion as described in Section 5.3.

**Figure 5.**
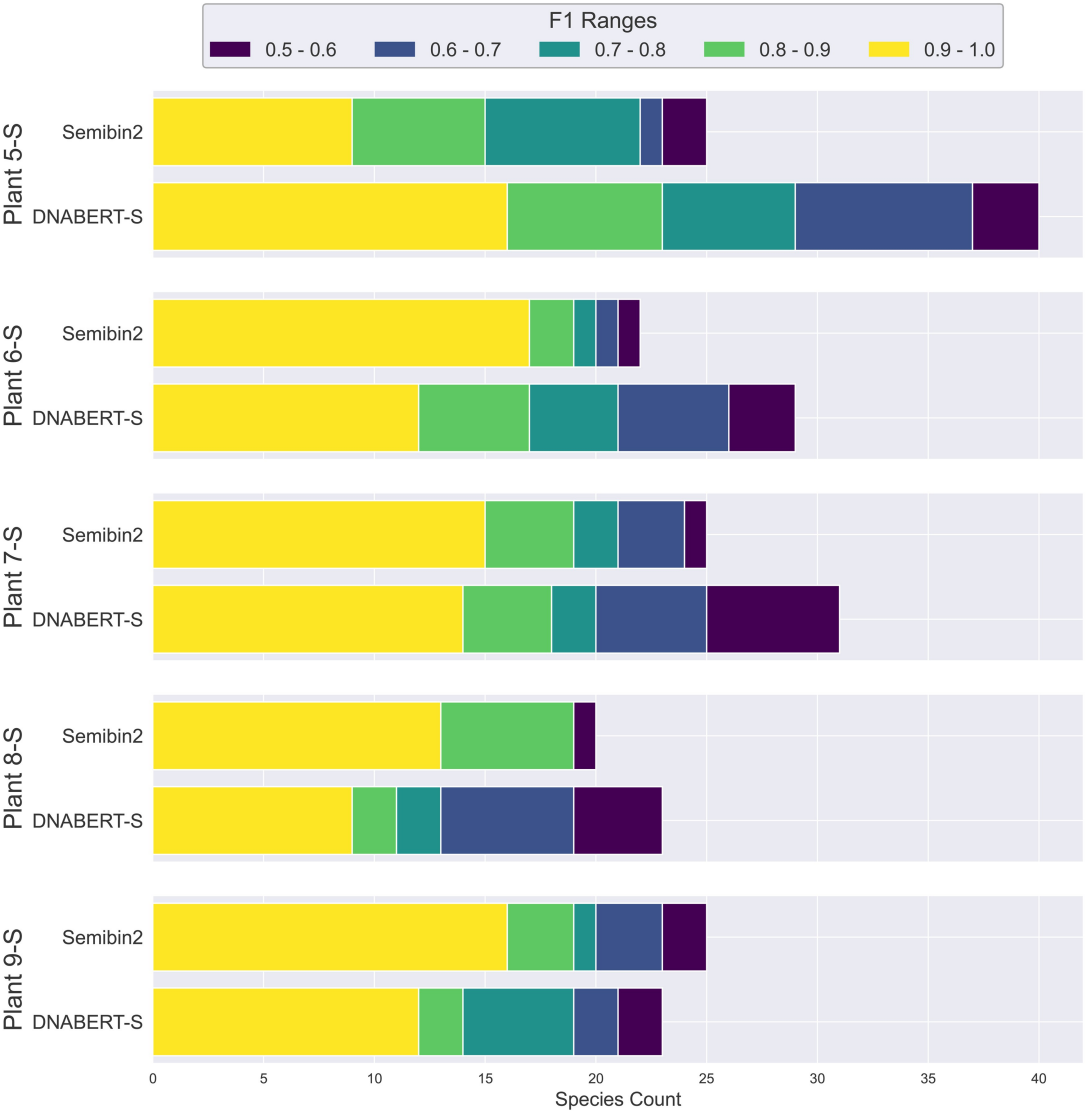
Compare DNABERT-S-based metagenomics binning algorithm with complete metagenomics binning tool SemiBin2. The bin size represents the number of unique species identified by each model, and different colors represent the F1 score of the identified species. With an F1 score >0.5, DNABERT-S identifies more species than SemiBin2 in 4/5 datasets.

As shown in Fig. 5, despite only using a portion of available information, the binning algorithm based on DNABERT-S embeddings achieves comparable or slightly better performance than SemiBin2, one of state-of-the-art complete metagenomics binning tool. This further demonstrates the effectiveness of DNABERT-S embedding in clustering and segregating sequences by species through DNA Embedding and shows its potential to further improve the area of metagenomics binning.

### 5.7 Selection of backbone model

This section delineates a comparative analysis of existing genome foundation models in the context of DNA embedding generation. We evaluated four renowned models: DNABERT (Ji *et al.* 2021), DNABERT-2 (Zhou *et al.* 2023), Nucleotide Transformer (Dalla-Torre *et al.* 2023), and HyenaDNA (Nguyen *et al.* 2023). Notably, DNABERT and the Nucleotide Transformer exhibit strict input sequence length limitations of 512 and 6144 (V1) or 12 288 (V2) base pairs, respectively. Conversely, DNABERT-2 and HyenaDNA do not impose such constraints. Considering the potentially extensive length of genome sequences in metagenomics binning, our preliminary experiments focused solely on DNABERT-2 and HyenaDNA. We start contrastive training from the pre-trained checkpoint of each model.

We train both models on our contrastive training datasets with the same set of hyperparameters for three epoch. We save checkpoints periodically and select the best checkpoint based on the models’ validation loss on the validation set. Since HyenaDNA, in general, requires larger learning rates than DNABERT-2, we train it with three different learning rates (3e−4, 3e−5, and 3e−6) and select the one that works best. For DNABERT-2, we only train it once with a learning rate of 3e−6. To avoid the impact of other factors, such as the schedule of curriculum learning, we train both models with the Weighted SimCLR loss only in the entire training process. We evaluate the models before and after contrastive training on our evaluation benchmark.

Table 3 presents the performance of both pre-trained DNABERT-2 and HyenaDNA, with and without contrastive training, in K-means clustering evaluations. DNABERT-2, despite underperforming without contrastive training, demonstrated superior performance compared to HyenaDNA post-training, highlighting its efficacy in learning effective DNA embeddings. Similar trends are observed in few-shot species classification scenarios, as detailed in Table 4. While DNABERT-2 initially exhibited subpar performance, it largely improved post-contrastive training, achieving the best results. Thus, DNABERT-2 was chosen as the backbone model for DNABERT-S.

**Table 3.** Performance of DNABERT-2 and HyenaDNA on K-Means clustering measured by Adjusted Rand Index (ARI).^a^

	Synthetic		Marine					Plant					Ave.
Dataset ID	0	1	0	1	2	3	4	0	1	2	3	4	
**DNABERT-2 w/o**	15.73	16.74	13.24	13.53	12.99	10.41	11.87	15.70	16.28	16.32	13.99	13.66	14.21
**DNABERT-2 w/**	**69.33**	**68.37**	**53.18**	**51.94**	**51.91**	**46.60**	**49.69**	**49.05**	**50.33**	**49.68**	**48.57**	**49.83**	**50.08**
Δ	53.61	51.63	39.94	38.41	38.93	36.19	37.82	33.35	34.05	33.36	34.57	36.17	39.00
**HyenaDNA w/o**	20.04	18.99	16.54	16.64	16.47	13.35	14.85	24.06	25.33	26.18	21.01	21.16	19.55
**HyenaDNA w/**	57.58	55.19	42.92	42.68	42.24	36.17	40.10	41.42	40.36	40.46	36.87	38.25	42.85
Δ	37.54	36.20	26.38	26.04	25.77	22.82	25.24	17.36	15.03	14.28	15.86	17.09	23.30

a Δ : the model’s performance improvement after contrastive training.

**Table 4.** Performance of DNABERT-2 and HyenaDNA on few-shot classification measured by Macro F1 score.^a^

	Synthetic				Marine				Plant				Ave.
Num Shots	1	2	5	10	1	2	5	10	1	2	5	10	
**DNABERT-2 w/o**	24.43	34.81	48.93	58.58	19.50	28.45	40.64	48.98	21.04	28.16	38.50	45.46	36.46
**DNABERT-2 w/**	**72.02**	**78.63**	**84.55**	**86.99**	**48.23**	**57.90**	**64.80**	**67.94**	**44.97**	**52.35**	**60.35**	**64.63**	**65.28**
Δ	47.60	43.83	35.62	28.41	28.73	29.45	24.16	18.96	23.92	24.20	21.85	19.17	28.82
**HyenaDNA w/o**	30.13	41.18	54.86	64.03	23.92	33.94	47.47	55.50	28.15	36.97	48.20	55.24	43.30
**HyenaDNA w/**	59.58	67.79	74.62	78.53	43.60	53.70	62.00	65.55	43.46	52.12	59.40	62.80	60.26
Δ	29.45	26.62	19.76	14.50	19.68	19.76	14.52	10.05	15.31	15.15	11.20	7.55	16.96

a Δ : the model’s performance improvement after contrastive training.

## 6 Discussion

We introduce DNABERT-S, a model tailored for species-aware DNA embeddings, which is empowered by the proposed Manifold Instance Mixup (MI-Mix) training objective and the Curriculum Contrastive Learning (C^2^LR) strategy. We perform extensive experiments on 23 datasets across thousands of different species and a variety of challenging tasks, including species clustering, classification, and metagenomics binning, to demonstrate the DNABERT-S’s capability of species differentiation.

Furthermore, we conduct a series of experiments on the training objective, backbone model selection, impacts of sequence length, and feature dimension to provide empirical insights for DNA embedding learning. We also compare DNABERT-S with traditional techniques like MMSeq2 in the problem of species differentiation and demonstrate that DNABERT-S can achieve slightly better performance with less amount of labeled samples. We envision DNABERT-S to potentially change the way genomic problems are approached from an embedding perspective. The primary limitation of DNABERT-S lies in its high computational demands, a common trait among deep learning models, when compared to more traditional, lightweight methods like Tetranucleotide Frequencies (TNF).

To efficiently handle massive scale data, especially in cases where computing and memory are limited, one may further distill and quantize the model for embedding calculation while compressing the feature dimension as discussed in Supplementary Fig. S10 (Supplementary Appendix S9.7 in Supplementary Material).

## Supplementary Material

btaf188_Supplementary_Data

## Data Availability

Data is available at https://github.com/MAGICS-LAB/DNABERT_S.
