# Supplementary material for: DNABERT-S: pioneering species differentiation with species-aware DNA embeddings
Source: Bioinformatics. 2025 Jul 15;41(Suppl 1):i255–64. doi: 10.1093/bioinformatics/btaf188 (PMC12261423; doi:10.1093/bioinformatics/btaf188)
Supplement: btaf188_Supplementary_Data [file btaf188_supplementary_data.pdf]

7. Algorithm for Metagenomics Binning

Algorithm 1 describes the unsupervised clustering algorithm we used for metagenomics binning, where $\mathtt{s}(E_i, E_j)$ represents the cosine similarity of two vectors E_i and E_j. **Selection of threshold .** As shown in Algorithm 1, the threshold is the most important hyperparameter that greatly impacts the final binning results. A high threshold results in small and dense clusters while a low threshold results in large yet sparse clusters. Since different models generate embeddings with distinct distributions, a fixed threshold (e.g., 0.9) could be too high for one model yet too low for another one. In practice, massive hyperparameter searches are needed to determine the best threshold for each model on different datasets. Due to the large size of our experiments and the various types of models we used, an automatic way is needed to fairly choose the threshold for each model on each dataset. For each metagenomics binning dataset, we use the dataset from the same source (e.g., Marine) as it with ID 0 to compute a threshold for each model on it, Specifically, we generate embeddings for each DNA sequence in the dataset and compute the similarities between each DNA sequence and its species center (i.e., the average of all the DNA sequence belongs to this species). The 70 percentile of all the similarities is used as the threshold. **Other hyperparameters.** We set minimum bin size $m = 10$, number of steps $Z = 1000$, and number of iterations $T = 3$. We also experimented with $T = 3, 4, 5$ and $60, 70, 80, 90$ percentile of all the similarities is used as the threshold , and found that the results are robust to these hyperparameters.

Algorithm 1 Modified K-Medoid Clustering

Require: threshold , minimum bin size m, embeddings $\boldsymbol{E} \in \mathbb{R}^{N\times d}$, number of steps Z, number of iterations T
Initialize predictions $\boldsymbol{p} \in \mathbb{R}^N$, $p_i =$ 1 for $i = 1, \ldots, N$
Initialize similarity matrix $\boldsymbol{S} = \boldsymbol{E}\boldsymbol{E}^\top$ with $S_{ij} = 0$ if $S_{ij} <$
Initialize density vector $\boldsymbol{d} \in \mathbb{R}^N$ with $d_i = \sum_{j=1}^{N} S_{ij}$
for step $z = 1$ to Z **do**
  Select seed index $s = \arg\max_{s'} d_{s'}$ and corresponding seed E_s
  for iteration $t = 1$ to T **do**
    Find neighborhood indices $\mathcal{I}$ of E_s where $\mathtt{s}(E_i, E_s) >$ and $p_i =$ 1 for each $i \in$
    Update seed: E_s $\frac{1}{|\mathcal{I}|}\sum_{i\in\mathcal{I}} E_i$
  end for
  Set p_i z, d_i 0 for each $i \in$
  Set d_x d_x $\sum_{i\in\mathcal{I}} S_{xi}$ for each $x \in [1, 2, \ldots, N]$
end for
for step $z = 1$ to Z **do**
  Find indices $\mathcal{I}$ where $p_i = z$ for each $i \in$
  if $|\mathcal{I}| < m$ **then**
    Set p_i 1 for each $i \in$
  end if
end for
Ensure: predictions $\boldsymbol{p}$

8. Data Statistics of Evaluation Benchmark

This section details the comprehensive statistics of the 28 datasets utilized for evaluating various DNA embedding models, as summarized in Table 5. For tasks involving clustering and classification, each dataset encompasses between 93 to 499 distinct species. From each species, 100 DNA sequences are sampled. These sequences vary in length, ranging from 2,000 to 20,000 base pairs. In the case of metagenomics binning, the datasets exhibit an unbalanced distribution of sequences across different species. Specifically, the number of sequences per species varies significantly, ranging from as few as 1 to as many as 4,599.

Our benchmark contains assets from GenBank [1] (license: Creative Commons Attribution Non-Commercial License `http://creativecommons.org/licenses/by-nc/2.0/uk/`) and CAMI2 [19] (license: Creative Commons Attribution 4.0 International License `http://creativecommons.org/licenses/by/4.0/`). It is worth noting that both GenBank and CAMI2 preprocess the RNA sequences into DNA equivalents by replacing U with T. Thus, although we did not explicitly analyze RNA sequences, many RNA viruses are considered in both model training and evaluation.

9. More Experimental Results

In this section, we provide additional experimental analysis. In Sec. 9.1, we present the performance of the models on species classification using a linear regression model across 6 additional datasets not covered in Sec. 5.4. In Sec. 9.2, we present the results of our investigation into the non-linear descriptiveness of embeddings by conducting experiments using logistic regression or a non-linear multi-layer perceptron (MLP). In Sec. 9.3, we present detailed results on species clustering and few-shot classification on DNABERT-S and the most competitive baseline models. In Sec. 9.4, we validate the effectiveness of DNABERT-S in situations where abundant labeled data is available by comparing it with MMseqs2 [31]. In Sec. 9.5, we show the performance of DNABERT-S to distinguish genomics sequences from species that are largely different from the ones in the training set. In Sec. 9.6, we delve into the influence of DNA sequence length on the performance of DNABERT-S. In Sec. 9.7, we investigate how changes in embedding dimensions affect the performance of DNABERT-S. In Sec. 9.8, we evaluate the impact of species-aware embedding on other types of genomics analysis tasks, like genomics function prediction tasks.

Table 5. Data statistics of the datasets for the DNA embedding evaluation. This table presents the sampling source, ID, number of sequences, number of sequences, the minimum / maximum / medium values of the sequence lengths and number of sequences in each species, and sequence type. We use the same set of balanced datasets for clustering and classification and another set of datasets for metagenomics binning.

Tasks	Source	ID	Species	Sequences	Sequence Length	Num. Per Species	Sequence Type
	`Marine`	0	326	32600	2k / 20k / 7.6k	100 / 100 / 100	Long-read Assembly
**Unsupervised**	`Marine`	1	375	37500	2k / 20k / 8.2k	100 / 100 / 100	Long-read Assembly
**Clustering**	`Marine`	2	361	36100	2k / 20k / 8.5k	100 / 100 / 100	Long-read Assembly
**&**	`Marine`	3	499	49900	2k / 20k / 6.8k	100 / 100 / 100	Long-read Assembly
**Few-Shot**	`Marine`	4	360	36000	2k / 20k / 7.1k	100 / 100 / 100	Long-read Assembly
**Classification**	`Plant`	0	108	10800	2k / 20k / 6.6k	100 / 100 / 100	Long-read Assembly
	`Plant`	1	100	10000	2k / 20k / 6.4k	100 / 100 / 100	Long-read Assembly
	`Plant`	2	93	9300	2k / 20k / 6.2k	100 / 100 / 100	Long-read Assembly
	`Plant`	3	129	12900	2k / 20k / 5.5k	100 / 100 / 100	Long-read Assembly
	`Plant`	4	129	12900	2k / 20k / 5.7k	100 / 100 / 100	Long-read Assembly
**(Microbe)**	`Synthetic`	0	200	20000	10k / 10k / 10k	100 / 100 / 100	Reference Genome
	`Synthetic`	1	200	20000	10k / 10k / 10k	100 / 100 / 100	Reference Genome
**(Mammalian,**	`Synthetic`	2	210	21000	10k / 10k / 10k	100 / 100 / 100	Reference Genome
**Invertebrate,**	`Synthetic`	3	210	21000	10k / 10k / 10k	100 / 100 / 100	Reference Genome
**Protozoa)**	`Synthetic`	4	210	21000	10k / 10k / 10k	100 / 100 / 100	Reference Genome
	`Marine`	5	515	119465	2.5k / 20k / 4.3k	10 / 841 / 201	Long-read Assembly
	`Marine`	6	527	125194	2.5k / 20k / 4.4k	10 / 915 / 223	Long-read Assembly
	`Plant`	5	181	71642	2.5k / 20k / 3.7k	10 / 4293 / 190	Long-read Assembly
	`Plant`	6	196	68426	2.5k / 20k / 3.7k	10 / 4599 / 116	Long-read Assembly
**Metagenomics**	`Plant`	5-S	61	16424	2.5k / 20k / 4.2k	1 / 1087 / 56	Short-read Assembly
**Binning**	`Plant`	6-S	57	9891	2.5k / 20k / 5.4k	1 / 1061 / 43	Short-read Assembly
**(Microbe)**	`Plant`	7-S	54	13205	2.5k / 20k / 5.0k	1 / 1203 / 58	Short-read Assembly
	`Plant`	8-S	47	8163	2.5k / 20k / 5.1k	1 / 1056 / 40	Short-read Assembly
	`Plant`	9-S	50	9512	2.5k / 20k / 5.2k	1 / 1155 / 34	Short-read Assembly
	`Synthetic`	5	323	37278	10k / 10k / 10k	31 / 200 / 111	Reference Genome
	`Synthetic`	6	249	28206	10k / 10k / 10k	30 / 199 / 114	Reference Genome
**Classification**	`Synthetic`	7	200	95309	10k / 10k / 10k	452 / 600 / 600	Reference Genome
**(Microbe)**	`Synthetic`	8	200	95475	10k / 10k / 10k	442 / 600 / 600	Reference Genome

9.1. Remaining Results on Species Classification

In this section, we present the performance of the models on species classification using a linear regression model across 6 additional datasets not covered in Sec. 5.4. As shown in Figure 6, the results are consistent with those shown in Figure 4. We also provide detailed results for all baselines on all 12 datasets for completeness in Table 14.

9.2. Results Comparison with Linear and Non-Linear Classifiers

In this section, we present the results of our investigation into the non-linear descriptiveness of embeddings by conducting experiments using logistic regression or a non-linear multi-layer perceptron (MLP). Table 6 and 7 show the results for three datasets: “Marine 0”, “Plant 0”, and “Synthetic 0”. The results demonstrate that DNABERT-S consistently achieves the best performance.

Table 6. DNABERT-S’s performance of **species classification** with varying numbers of training samples on datasets “Marine 0” and “Plant 0”: Beyond using **logistic regression (LR)**, we also train a **multi-layer perceptron (MLP)** with non-linear activation function (ReLU). The term "**Difference**" denotes the performance gap between "DNABERT-S" and the "best-baseline". The results show that DNABERT-S embedding consistently outperforms the best-existing baseline in both linear and non-linear discriminativity.

	**Marine 0**					**Plant 0**				
**Dataset ID**	**1**	**2**	**5**	**10**	**20**	**1**	**2**	**5**	**10**	**20**
**LR: best-baseline**	27.65	38.81	52.4	58.86	63.29	28.15	36.97	48.2	55.24	60.04
**LR: DNABERT-S**	50.25	59.41	66.07	68.92	70.75	47.83	55.83	63.01	67.12	69.82
**LR: Difference**	**22.60**	**20.60**	**13.67**	**10.06**	**7.46**	**19.68**	**18.86**	**14.81**	**11.88**	**9.78**
**MLP: best-baseline**	26.07	38.59	53.86	60.27	63.07	27.39	36.19	47.5	54.03	59.13
**MLP: DNABERT-S**	48.55	59.25	66.09	68.95	69.99	45.31	55.34	63.25	67.25	70.00
**MLP: Difference**	**22.48**	**20.66**	**12.23**	**8.68**	**6.92**	**17.92**	**19.15**	**15.75**	**13.22**	**10.87**

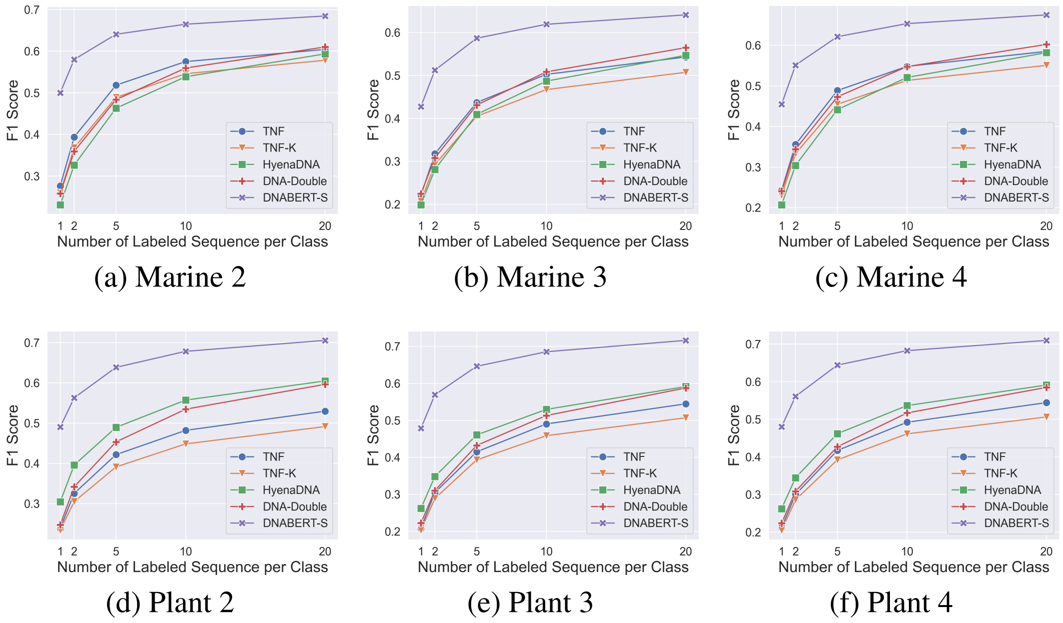

Fig. 6. Results of species classification using linear regression on other 6 datasets.

Table 7. DNABERT-S's performance of **species classification** with varying numbers of training samples on dataset "Synthetic 0": Beyond using **logistic regression (LR)**, we also train a **multi-layer perceptron (MLP)** with non-linear activation function (ReLU). The term "**Difference**" denotes the performance gap between "DNABERT-S" and the "best-baseline". The results show that DNABERT-S embedding consistently outperforms the best-existing baseline in both linear and non-linear discriminativity.

	Synthetic 0				
**Dataset ID**	**1**	**2**	**5**	**10**	**20**
**LR: best-baseline**	44.07	56.11	68.69	75.34	79.54
**LR: DNABERT-S**	71.36	77.93	83.37	85.81	87.77
**LR: Difference**	**27.29**	**21.82**	**14.68**	**10.47**	**8.23**
**MLP: best-baseline**	40.28	54.95	69.59	76.54	81.33
**MLP: DNABERT-S**	68.96	77.47	83.44	85.86	87.77
**MLP: Difference**	**28.68**	**22.52**	**13.85**	**9.32**	**6.44**

9.3. Results with Error Bars

In this section, we present detailed results on species clustering and few-shot classification on DNABERT-S and the most competitive baseline models. Table 8, 9, and 10 respectively show the models' mean and std on each setting across 5 random seeds. As shown in the tables, DNABERT-S consistently outperforms the baselines with small variances.

Table 8. Performance of models with error bars on "Synthetic 0" dataset. We evaluate models using K-Means clustering (Sec. 5.2) and 1/2/5/10/20-shot classification (Sec. 5.4).

	Synthetic 0					
	**ARI**	**1**	**2**	**5**	**10**	**20**
**TNF**	38.18 ± 1.27	44.30 ± 0.97	56.13 ± 1.10	68.68 ± 0.73	75.24 ± 0.37	79.48 ± 0.07
**TNF-K**	36.11 ± 0.09	39.51 ± 0.35	50.26 ± 0.89	62.43 ± 0.41	68.53 ± 0.50	72.95 ± 0.36
**HyenaDNA**	20.10 ± 0.50	30.03 ± 0.49	41.21 ± 0.92	54.42 ± 0.73	63.79 ± 0.59	70.53 ± 0.25
**DNA-Double**	34.91 ± 0.90	34.61 ± 0.91	46.79 ± 0.39	59.92 ± 0.53	67.45 ± 0.20	73.64 ± 0.25
**DNABERT-S**	66.94 ± 2.07	71.54 ± 0.51	77.77 ± 0.68	83.12 ± 0.18	85.63 ± 0.18	87.68 ± 0.15

9.4. Comparison with alignment-based method

While previous experiments have highlighted DNABERT-S's exceptional performance in scenarios with limited or no labeled data, this section focuses on its effectiveness in situations where abundant labeled data is available. Specifically, we aim to understand the embedding-based species differentiation method in scenarios where reference genomes of the species to classify are available. We compare embedding-based methods with MMseqs2 [31], a leading alignment-based species classification tool.

Table 9. Performance of models with error bars on "Marine 0" dataset. We evaluate models using K-Means clustering (Sec. 5.2) and 1/2/5/10/20-shot classification (Sec. 5.4).

	Marine 0					
	**ARI**	**1**	**2**	**5**	**10**	**20**
**TNF**	24.78 ± 0.23	27.89 ± 0.95	38.69 ± 0.04	52.36 ± 0.26	58.95 ± 0.05	62.69 ± 0.10
**TNF-K**	25.44 ± 0.50	22.82 ± 0.35	30.06 ± 0.61	40.50 ± 0.14	45.20 ± 0.25	49.35 ± 0.15
**HyenaDNA**	16.31 ± 0.07	23.89 ± 0.83	33.59 ± 0.01	47.50 ± 0.39	55.61 ± 0.18	61.75 ± 0.13
**DNA-Double**	26.82 ± 0.45	26.87 ± 1.16	36.98 ± 0.21	49.86 ± 0.12	57.93 ± 0.26	63.33 ± 0.06
**DNABERT-S**	53.91 ± 0.22	50.37 ± 0.74	59.71 ± 0.11	66.03 ± 0.11	69.00 ± 0.19	70.75 ± 0.13

Table 10. Performance of models with error bars on "Plant 0" dataset. We evaluate models using K-Means clustering (Sec. 5.2) and 1/2/5/10/20-shot classification (Sec. 5.4).

	Plant 0					
	**ARI**	**1**	**2**	**5**	**10**	**20**
**TNF**	26.10 ± 0.70	24.07 ± 0.40	32.04 ± 1.00	43.35 ± 0.24	48.92 ± 0.27	53.29 ± 0.39
**TNF-K**	25.83 ± 0.67	22.82 ± 0.35	30.06 ± 0.61	40.50 ± 0.14	45.20 ± 0.25	49.35 ± 0.15
**HyenaDNA**	24.61 ± 0.77	28.45 ± 0.30	36.46 ± 0.61	48.56 ± 0.30	55.13 ± 0.53	59.80 ± 0.27
**DNA-Double**	22.10 ± 0.46	24.80 ± 0.56	32.91 ± 0.52	44.82 ± 0.18	52.77 ± 0.48	59.35 ± 0.37
**DNABERT-S**	51.15 ± 1.13	48.39 ± 1.33	55.92 ± 0.83	62.97 ± 0.36	67.14 ± 0.41	69.64 ± 0.15

For a fair comparison with MMseqs2, which relies on the reference genomes of each species when performing classification, we construct two datasets, each consisting of 200 distinct species. To mimic real-world setups, instead of classifying segment of reference genomes, we simulate 600 long-reads with PBSIM2 [24] from each selected species, 100 as the test set and 500 as the training set. For the embedding-based methods, such as DNABERT-S and TNF, we generate embedding for all these sequences and use K-Neareast-Neighbor (KNN) classifier with n equals to 5 for species classification. We respectively use 100, 200, 300, 400, and 500 sequences from each species to construct the training set. For the MMseqs2, we respectively use the reference genome and the simulated long-read sequences as the reference for alignment-based classification.

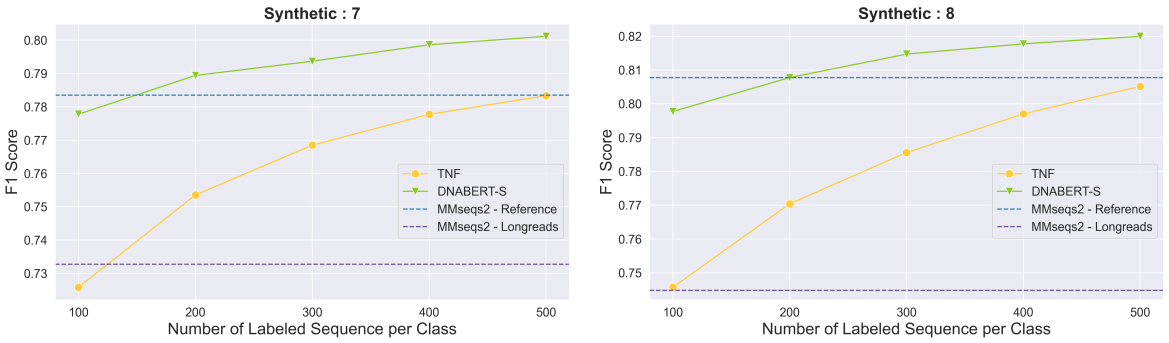

(a) Synthetic 7 (b) Synthetic 7

Fig. 7. Results on species classification when reference genomes are available.

Figure 7 the results of the models on the datasets. As shown in the figure, DNABERT-S starts to outperforms MMseqs2 with 200-300 labeled sequence from each species with a simple KNN classier, while TNF achieves comparable performance as MMseqs2 with about 500 labels sequence per species. These results indicate the potential of embedding-based methods to replace traditional alignment-based method in species classification in data abundant scenarios. Yet a fact that cannot be ignored is that DNABERT-S is much more computational cost than MMseqs2. As a comparison, MMseqs2 requires about 30 seconds on a single CPU to make predictions while DNABERT-S requires about 1 hour on 2 NVIDIA A100 GPUs to do the same thing. Therefore, there is a long way to go to fully replace alignment-based methods in high-throughput genomics analysis.

9.5. Results on Non-Microbe Species

Since DNABERT-S is trained on microbe species (e.g., viruses, fungi, and bacteria), a natural question is whether it can distinguish genomics sequences from species that are largely different from the ones in the training set. To answer this question, we construct 3 synthetic datasets (ID: 2, 3, and 4) that include genomes from invertebrate, protozoa, and mammalian species. We randomly select 70 species from each category to achieve 210 species in total. We perform the same clustering and few-shot classification as presented in Table 1 and Figure 4.

As shown in Table 11, DNABERT-S consistently outperforms baselines across all the datasets and evaluation scenarios, indicating DNABERT-S's transferability and robustness on species that are significantly different from its training set. However, the improvements over the baselines are less significant, and the absolute scores, such as ARI in clustering and F1 in classification, are also lower than those in microbe datasets. On the one hand, there are higher genetic similarities among

Table 11. Performance of models on non-microbe species. We evaluate models on each dataset using K-Means clustering (Sec. 5.2) and 1/5/20-shot classification (Sec. 5.4).

	Synthetic:2				Synthetic:3				Synthetic:4			
**Dataset ID**	**ARI**	**1**	**5**	**20**	**ARI**	**1**	**5**	**20**	**ARI**	**1**	**5**	**20**
**TNF**	20.70	26.28	45.71	56.24	19.11	25.05	42.69	52.27	21.49	25.94	44.96	54.60
**TNF-K**	18.63	22.42	40.07	50.75	16.91	20.38	36.48	46.74	19.30	21.38	39.23	48.94
**HyenaDNA**	11.20	17.29	34.77	48.76	10.86	16.50	32.69	45.26	11.58	17.30	35.38	47.86
**DNABERT-2**	9.18	15.91	33.75	49.00	8.99	15.28	31.03	45.17	9.79	15.38	33.75	47.78
**DNA-Double**	13.01	16.04	30.56	42.11	12.80	15.91	28.82	39.28	14.15	16.20	31.22	42.25
**DNABERT-S**	**32.70**	**33.21**	**49.78**	**59.01**	**29.44**	**29.78**	**45.65**	**54.63**	**33.60**	**32.58**	**49.67**	**57.97**

mammals compared to the often more significant genetic diversity found in microbes, making it more challenging to distinguish different mammalian species. On the other hand, due to the significant distinction between microbe and mammalian/protozoa species, some of the differentiation rules and markers learned from microbe genomes may not be applicable to genomes of species in other categories, which also suggests the needs of in-domain species-aware training.

9.6. Impact of Sequence Length

This section delves into the influence of DNA sequence length on final model performance, examined from both training and evaluation standpoints.

9.6.1. Varying Sequence Length in Training

Training with longer DNA sequences increases the need for more memory and computing power. It also means we can only use smaller batches of data at a time. Therefore, the length of the sequences is an important factor in contrastive training as it affects how much it costs to train the model. To see how different sequence lengths affect training, we did three experiments using the same data. Our training data has sequences that are 10000bp long. For experiments with shorter sequences S, we only used the first S nucleotides of each DNA sequence. We tested sequence lengths of 500bp, 2000bp, and 10000bp, training only with Weighted SimCLR loss and starting from the pre-trained DNABERT-2 model.

Figure 8 shows the results for the three models, along with the pre-trained DNABERT-2 without contrastive training and the strongest baseline, TNF. The findings reveal that sequence length significantly influences the model's performance. Training even on short sequences, such as 500bp, leads to substantial improvements. The model trained with 500bp sequences performs nearly as well as TNF. When we increase the input sequence length from 500bp to 2000bp, there's a marked improvement in performance. A similar trend is observed when increasing the sequence length from 2000bp to 10000bp. These results highlight the importance of sequence length in training an effective model. Therefore, we decided to train our model with 10000bp sequences, despite the higher computational requirements.

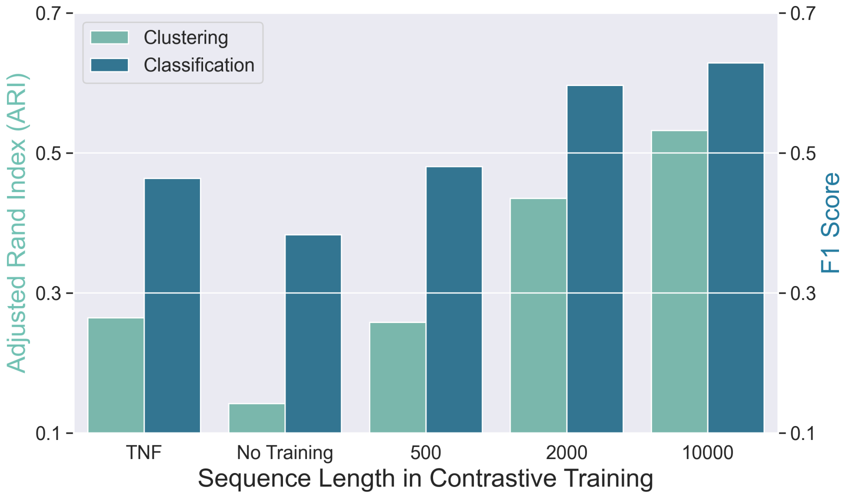

Fig. 8. Performance of DNABERT-S in clustering and classification with different sequence lengths used in contrastive training.

9.6.2. Varying Sequence Length in Evaluation

In this part, we assess how the length of DNA sequences in evaluation impacts performance. We use two synthetic datasets for clustering and classification tasks. Each sequence in these datasets is deliberately constructed to be 10000bp long. This allows us to create a test set where all sequences have the same length. We test sequence lengths ranging from 32bp (2^5) to 8192bp (2^{13}). For each test with different sequence lengths, we keep everything else the same, like how we split the data into training and testing sets and the settings for logistic regression.

Figure 9 presents the performance of TNF and DNABERT-S with various sequence lengths. The results show that both models significantly benefit from longer sequences. When the sequence length is less than 256bp (2^8), both models perform poorly in clustering and classifying samples. However, as the sequence length increases, starting from 512bp (2^9), DNABERT-S begins to outperform TNF. The performance gap between the two models gets bigger as the sequence length increases. These findings highlight the crucial role of sequence length in effectively differentiating between species.

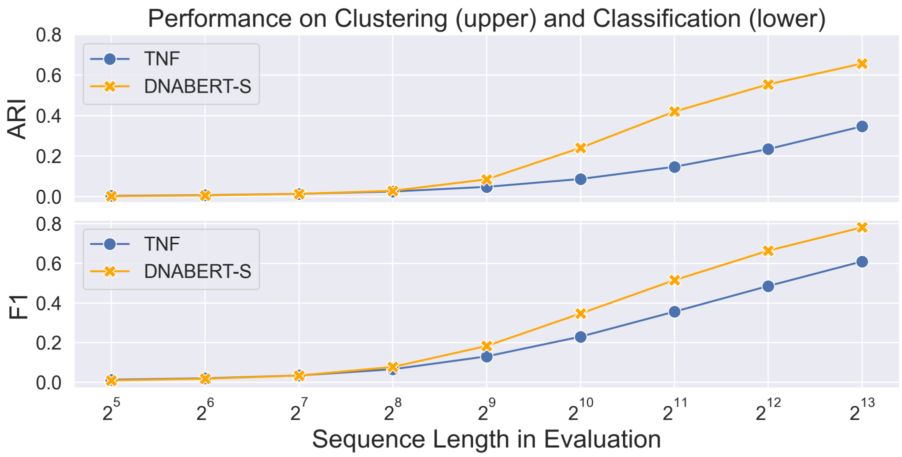

Fig. 9. Performance of DNABERT-S and TNF on clustering (upper) and classification (lower) with different input sequence lengths during evaluation.

9.7. Impact of Embedding Dimension Reduction

This section investigates how changes in embedding dimensions affect the performance of DNABERT-S, a key aspect influencing the scalability of DNA embeddings generated by the model. Initially, DNA embeddings for all clustering and classification datasets are computed using the trained DNABERT-S. To reduce embedding dimensions, we use an average pooling layer with a consistent kernel size and stride S. This process effectively averages S consecutive dimensions into one new dimension. We test with S values of 96, 48, 24, 12, 6, 3, and 2, corresponding to reduced embedding dimensions of 8, 16, 32, 64, 128, 256, and 384, respectively.

Figure 10 illustrates DNABERT-S's performance with these varying feature dimensions, in comparison to TNF. The results demonstrate that DNABERT-S's embedding is quite resilient to dimension compression. It maintains nearly the same performance level even when reduced to 256 dimensions and only experiences a notable drop in performance when compressed to 32 dimensions. Notably, DNABERT-S still surpasses the 256-dimensional TNF feature even when its own dimensionality is reduced to just 16. This robustness to dimension reduction enhances its practical applicability in various genomic contexts.

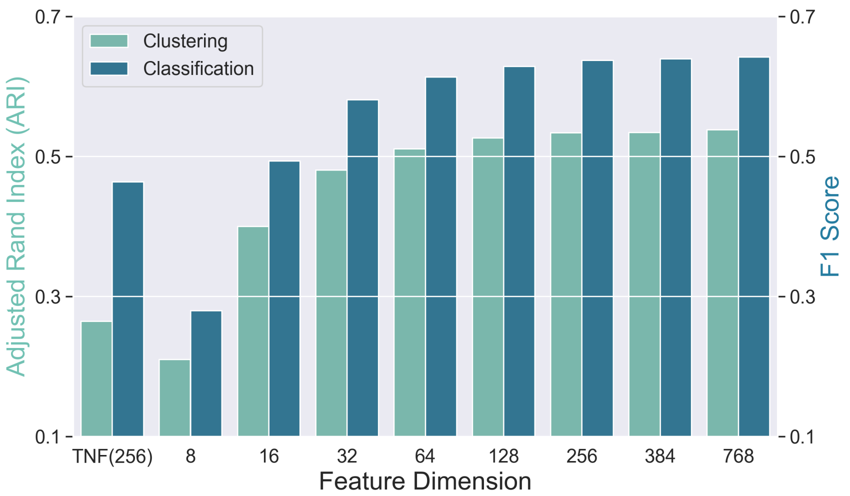

Fig. 10. DNABERT-S's performance with varying embedding dimensions reduced by average pooling. DNABERT-S is robust to feature dimension reduction, and it even outperforms TNF with 16-dimensional embedding.

9.8. Species-Aware Embeddings on Genomics Function Predictions

This section evaluates the impact of species-aware embedding on other types of genomics analysis tasks. Specifically, we aim to understand how the species-aware embeddings perform compared to the embedding generated by the genome foundation model without contrastive training on various types of genomics function prediction tasks. We utilize the GUE benchmark [36], which comprises a comprehensive collection of 28 datasets covering 7 diverse tasks, such as epigenetic marks prediction, promoter prediction, and transcription factor binding site prediction. Following our established methodology, each model is respectively used to generate embeddings for each DNA sequence, and a logistic regression model is trained for classification. The Matthews Correlation Coefficient (MCC) serves as the evaluation metric. We perform experiments on both DNABERT-2 and HyenaDNA.

The results, as detailed in Table 12, show that after species-aware conservative training, DNABERT-S underperforms DNABERT-2 on 19 of the 28 datasets, with an average loss of 2.12 in the MCC. Similarly, on HyenaDNA, the one went through species-aware training underperforms the original one on 18 out of 28 datasets. As a model honed for species-aware tasks, DNABERT-S may exhibit reduced generalizability compared to broader genome foundation models (e.g., DNABERT-2). However, this specialized focus should not be viewed as a disadvantage. The intrinsic design of DNABERT-S—to prioritize species-specific features—may naturally limit its applicability to a broader range of tasks, a trade-off inherent to its specialized nature. For instance, when considering sequences from different species with varying functions, DNABERT-S's training objective emphasizes species-specific similarities over functional commonalities, a choice that is deliberate for its targeted application.

10. i-Mix for Original Contrastive Learning

The Weighted SimCLR method treat each instance $\{x_i\}_{i=1}^{B}$ and $\{x_{i+}\}_{i=1}^{B}$ as anchors, with each anchor associated with $2B \quad 2$ negative samples. This section explains why integrating the i-Mix method into Weighted SimCLR (Sec. 3.1) doubles memory or training time.

For clarity, we define $x_{B+i} = x_{i+}$. We also expand the virtual labels v_i, which are $2B$-dimensional, to identify the positive sample for each anchor. Here $v_{i,\tilde{i}} = 1$ and $v_{i,j\neq\tilde{i}} = 0$, $\tilde{i} = (B+i)$ mod $2B$.

When the i-Mix method treats every sample from $\{x_i\}_{i=1}^{2B}$ as anchor, it begins by shuffling $\{(x_i, v_i)\}_{i=1}^{2B}$ to generate $\{(\hat{x}_i, \hat{v}_i)\}_{i=1}^{2B}$. For each anchor (x_i, v_i), it uses a simple mixup method to mix it up with $(\hat{x}_i, \hat{v}_i)$ before encoder layers of the model, using mixup coefficient $\quad_i \sim$ Beta(α, α). This mixing results in:

$$(h_i^0, v_i^{mix}) = (\quad_i x_i + (1 \quad \quad_i)\hat{x}_i, \quad_i v_i + (1 \quad \quad_i)\hat{v}_i).$$

Moreover, for each anchor x_i, if i-Mix considers all samples from $\{x_j\}_{j=1}^{2B} \backslash \{x_i\}$ as either positive or negative samples, the model $f(\cdot)$ must process both the initial data instances $\{x_i\}_{i=1}^{2B}$ and mixed data instances $\{h_i^0\}_{i=1}^{2B}$ to generate their embeddings. Therefore, the i-Mix requires nearly twice more memory or training time compared to the method in Sec. 3.1 if using the same batch size.

Table 12. Performance of DNABERT-2 and HyenaDNA before and after species-aware contrastive training on the GUE benchmark.

	Epigenetic Marks Prediction					
	**H3**	**H3K14ac**	**H3K36me3**	**H3K4me1**	**H3K4me2**	**H3K4me3**
**DNABERT-2**	66.87	**38.92**	**43.39**	31.79	**30.16**	**23.68**
**DNABERT-S**	**69.02**	37.45	41.91	**32.76**	27.86	22.16
**HyenaDNA w/o**	69.54	30.62	**38.95**	**33.29**	**30.90**	21.24
**HyenaDNA w/**	**69.67**	**32.06**	38.61	32.29	30.32	**23.21**

	Epigenetic Marks Prediction				Promoter Detection		
	**H3K79me3**	**H3K9ac**	**H4**	**H4ac**	**all**	**notata**	**tata**
**DNABERT-2**	57.01	**47.52**	73.75	35.54	78.31	40.25	**88.85**
**DNABERT-S**	**58.41**	44.70	**75.96**	**35.64**	**78.93**	**40.59**	88.62
**HyenaDNA w/o**	52.33	44.27	**72.93**	29.37	**77.06**	**53.73**	**86.25**
**HyenaDNA w/**	**54.99**	**44.45**	72.61	**29.85**	76.56	45.17	85.14

	Transcription Factor Prediction (Human)					Core Promoter Detection		
	**0**	**1**	**2**	**3**	**4**	**all**	**notata**	**tata**
**DNABERT-2**	**62.67**	**68.58**	**54.44**	**35.67**	**62.89**	**57.33**	**46.18**	61.48
**DNABERT-S**	60.34	65.28	47.75	30.54	59.45	56.52	39.05	**61.87**
**HyenaDNA w/o**	**61.37**	**65.96**	**45.97**	35.76	**58.32**	**56.14**	40.94	**60.31**
**HyenaDNA w/**	59.10	65.02	45.47	**36.68**	53.20	55.52	**42.29**	60.30

	Transcription Factor Prediction (Mouse)					Virus	Splice	
	**0**	**1**	**2**	**3**	**4**	**Covid**	**Reconstruct**	Ave.
**DNABERT-2**	**37.08**	69.56	**67.71**	**42.26**	**34.80**	**54.50**	**24.85**	**51.28**
**DNABERT-S**	31.38	**71.13**	56.71	39.85	28.94	49.84	23.87	49.16
**HyenaDNA w/o**	**23.48**	**58.28**	54.88	21.34	**22.15**	30.50	**28.71**	**46.59**
**HyenaDNA w/**	21.24	53.88	**55.54**	**23.86**	18.66	**31.08**	22.11	45.67

11. Comparison of Parameters, Embedding Dimensions, Inference Time, and Memory

In this section, we compare the number of parameters (million), embedding dimensions, inference time (seconds), and inference memory (MB) for all models listed in Table 1. We show the results in Table 13.

Table 13. Comparison of the number of parameters (million), embedding dimensions (million), inference time (seconds), and memory (MB) for all models listed in Table 1. The symbol "-" denotes that the inference time or memory is negligible.

Model	Num. Params (M)	Emb. Dim.	Inf. Time (Sec.)	Inf. Mem. (MB)
**TNF**	0	256	-	-
**TNF-K**	0.026	768	-	-
**TNF-VAE**	3	103	-	-
**DNA2Vec**	0.026	100	-	-
**HyenaDNA**	28.2	256	11.16	995
**Hyena-Sim**	28.2	256	11.16	995
**NT-v2**	97.9	512	19.16	1273
**DNABERT-2**	117	768	14.27	3991
**DNA-Dropout**	117	768	14.27	3991
**DNA-Double**	117	768	14.27	3991
**DNA-Mutate**	117	768	14.27	3991
**DNA-Sim**	117	768	14.27	3991
**DNABERT-S**	117	768	14.27	3991

12. Estimate Potential Data Leakage

We consider data leakage to occur when the same species are present in both training and evaluation datasets. As a large of species in evaluation datasets are unknown, there is no clear way to accurately analyze the overlapping between our training and evaluation dataset at the species level. Thus, we validate the absence of data leakage issues at the sequence level.

Our experiments utilize two categories of data: synthetic and CAMI2 datasets. For synthetic data, the data construction method ensures the absence of data leakage. We design the synthetic datasets to exclude any species present in the training data, thereby preventing potential data leakage. For the CAMI2 datasets, due to discrepancies in species annotations between CAMI2 and GenBank, direct validation was challenging. Therefore, we perform an alignment-based estimation using minimap2 [18]. We align each evaluation dataset to the training data and considered sequences with over 90% alignment to the training sequences as present in the training data.

We compute the presence rate as number of presented sequences$/$total number of sequences. It's important to note that different species can share common or highly similar genome sequences, so a non-zero presence rate is expected in real-world scenarios. As a reference, the two synthetic datasets with non-overlapping species have presence rates of 6.88% and 8.92%. For the CAMI2 datasets, the plant-associated ones have presence rates between 3.51% and 4.98%, which are even lower than the synthetic datasets. The marine datasets have presence rates between 7.99% and 9.45%, comparable to the synthetic ones. Based on these statistics, there is negligible species leakage between our training and evaluation data. Together with the experiments on synthetic datasets with absolutely no species leakage, we show DNABERT-S's compatibility in generalizing to unseen species.

Table 14. Model's performance on species classification using linear regression with varying numbers of training samples on all the 12 datasets.

Model	Synthetic 0					Marine 0					Plant 0				
	1	2	5	10	20	1	2	5	10	20	1	2	5	10	20
**TNF**	44.07	56.11	68.69	75.34	79.54	27.65	38.81	52.4	58.86	62.59	24.01	32.69	43.39	48.99	53.29
**TNF-K**	39.06	50.22	62.52	68.55	72.82	25.97	36.47	49.15	55.44	59.26	22.83	30.58	40.55	45.57	49.58
**TNF-VAE**	34.23	47.06	61.44	69.31	75.02	23.72	34.02	47.00	53.88	58.59	20.63	28.80	39.38	45.96	51.10
**DNA2Vec**	35.85	46.98	61.54	69.64	75.26	24.56	34.04	47.79	55.36	60.11	23.98	31.35	41.46	47.41	51.96
**HyenaDNA**	30.13	41.18	54.86	64.03	70.69	23.92	33.94	47.47	55.50	61.42	28.15	36.97	48.20	55.24	60.04
**DNABERT-2**	24.43	34.81	48.93	58.58	65.98	19.50	28.45	40.64	48.98	55.67	21.04	28.16	38.50	45.46	51.99
**DNA-Dropout**	21.09	29.39	40.80	48.38	54.44	15.42	21.47	30.99	38.05	44.06	19.05	24.78	33.12	38.99	44.30
**DNA-Double**	34.54	46.54	59.86	67.44	73.60	26.76	36.84	49.98	57.68	63.29	24.56	33.09	45.06	52.91	59.57
**DNA-Mutate**	21.27	29.92	41.78	50.31	57.20	15.70	21.74	31.86	39.32	45.95	18.16	24.40	33.58	40.23	46.09
**Hyena-Sim**	59.58	67.79	74.62	78.53	81.42	43.60	53.70	62.00	65.55	68.19	43.46	52.12	59.40	62.80	66.22
**DNA-Sim**	72.02	78.63	84.55	86.99	88.93	48.23	57.90	64.80	67.94	70.23	44.97	52.35	60.35	64.63	68.18
**DNABERT-S**	71.36	77.93	83.37	85.81	87.77	50.25	59.41	66.07	68.92	70.75	47.83	55.83	63.01	67.12	69.82

Model	Synthetic 1					Marine 1					Plant 1				
	1	2	5	10	20	1	2	5	10	20	1	2	5	10	20
**TNF**	43.16	54.76	68.15	74.75	78.82	26.42	38.30	51.65	57.82	60.94	24.21	33.42	44.14	50.05	54.74
**TNF-K**	37.69	48.42	61.66	68.15	72.30	24.43	35.61	47.97	54.25	57.92	23.45	31.42	41.34	47.00	51.08
**TNF-VAE**	33.87	46.13	60.72	68.70	74.20	22.93	33.27	45.89	52.89	57.41	20.43	29.19	39.88	46.91	52.02
**DNA2Vec**	35.00	46.13	61.20	69.44	74.55	24.17	33.63	47.06	54.29	58.45	24.03	31.81	41.93	48.29	52.97
**HyenaDNA**	29.56	40.52	55.49	64.09	70.10	23.27	33.17	47.45	55.70	61.42	29.93	39.06	49.09	55.59	60.73
**DNABERT-2**	23.87	34.01	48.29	57.24	64.39	19.06	27.41	40.19	48.69	55.52	22.02	29.70	40.11	47.59	53.34
**DNA-Dropout**	20.42	27.69	39.47	46.76	53.15	14.82	21.02	30.54	37.88	44.06	19.42	25.55	34.35	40.38	45.58
**DNA-Double**	33.82	44.25	57.85	66.31	72.54	26.41	36.51	49.72	57.24	62.80	23.93	33.28	45.28	53.16	59.45
**DNA-Mutate**	21.09	29.04	41.47	49.50	56.18	14.95	21.24	31.54	39.21	45.69	18.65	25.02	34.22	40.38	45.58
**Hyena-Sim**	57.58	66.16	74.08	77.74	80.49	42.86	53.40	61.51	65.27	67.65	46.35	54.27	60.39	63.79	66.66
**DNA-Sim**	70.54	77.96	83.78	86.41	88.38	47.71	56.62	63.68	67.09	69.35	46.85	54.57	61.50	65.19	68.63
**DNABERT-S**	69.30	77.13	82.88	85.35	87.06	49.42	58.12	64.94	67.85	69.95	49.82	57.62	64.14	67.24	69.81

Model	Marine 2					Marine 3					Marine 4				
	1	2	5	10	20	1	2	5	10	20	1	2	5	10	20
**TNF**	27.63	39.37	51.84	57.51	60.41	22.11	31.74	43.71	50.26	54.31	24.28	35.58	48.86	54.81	58.49
**TNF-K**	25.97	36.80	48.90	54.59	57.82	20.71	29.26	40.49	46.72	50.73	23.03	33.31	45.48	51.36	55.06
**TNF-VAE**	24.10	34.20	46.27	52.85	56.98	19.23	27.58	38.88	45.90	50.74	21.20	31.16	43.00	49.83	54.51
**DNA2Vec**	24.37	33.77	46.83	54.06	58.12	20.41	28.16	40.03	47.09	51.77	22.43	31.17	44.49	51.45	56.07
**HyenaDNA**	23.09	32.63	46.30	53.86	59.36	19.88	28.11	40.94	48.68	54.70	20.72	30.42	44.16	52.09	58.20
**DNABERT-2**	18.11	26.57	39.05	47.18	53.46	16.04	22.95	34.17	42.03	48.92	17.23	25.27	37.72	45.97	52.73
**DNA-Dropout**	14.93	21.06	30.01	37.00	42.79	12.55	17.61	25.63	31.77	37.30	13.40	18.94	28.23	34.69	40.79
**DNA-Double**	25.81	35.94	48.36	55.96	61.02	22.47	30.78	43.11	50.84	56.48	24.06	34.41	47.32	54.76	60.24
**DNA-Mutate**	14.88	20.85	30.70	38.07	44.09	12.57	17.50	26.18	32.84	38.90	13.97	19.56	29.13	36.23	42.60
**Hyena-Sim**	42.75	52.24	59.92	63.25	65.65	37.91	46.51	54.87	58.80	61.40	40.18	49.99	58.34	61.98	64.83
**DNA-Sim**	48.29	56.24	62.81	65.75	67.84	41.18	49.82	57.59	61.19	63.63	43.72	53.90	60.89	64.40	66.63
**DNABERT-S**	50.01	58.01	64.06	66.49	68.46	42.77	51.23	58.68	61.93	64.12	45.50	55.12	62.13	65.36	67.51

Model	Plant 2					Plant 3					Plant 4				
	1	2	5	10	20	1	2	5	10	20	1	2	5	10	20
**TNF**	23.83	32.52	42.16	48.19	52.96	20.85	30.49	41.50	49.03	54.45	21.18	30.01	41.71	49.21	54.43
**TNF-K**	23.36	30.48	39.10	44.83	49.13	20.31	28.90	39.32	45.85	50.69	20.49	28.59	39.21	46.13	50.61
**TNF-VAE**	19.86	28.90	39.24	45.67	51.14	18.42	26.50	37.51	44.62	51.14	18.46	26.85	38.17	45.50	51.26
**DNA2Vec**	24.10	31.57	41.04	47.42	52.14	21.49	29.63	40.20	47.19	52.53	21.92	29.43	40.22	47.70	52.56
**HyenaDNA**	30.44	39.57	48.93	55.71	60.47	26.22	34.83	46.07	52.96	59.12	26.23	34.46	46.17	53.64	59.10
**DNABERT-2**	22.36	29.75	40.32	47.50	53.33	18.75	25.90	36.95	44.80	51.66	19.42	25.95	36.98	44.96	51.33
**DNA-Dropout**	19.35	26.29	34.47	40.20	45.38	16.50	22.20	30.57	36.74	42.07	16.55	22.62	30.52	37.32	42.62
**DNA-Double**	24.72	34.16	45.32	53.45	59.63	22.24	31.03	43.23	51.30	58.74	22.35	30.85	42.68	51.68	58.47
**DNA-Mutate**	18.76	25.74	34.48	41.57	46.82	15.94	21.69	30.22	36.80	42.93	15.78	21.58	30.78	37.57	43.55
**Hyena-Sim**	45.45	52.78	60.04	63.88	67.19	41.27	50.02	58.21	62.80	66.03	42.14	50.28	58.68	63.51	66.41
**DNA-Sim**	45.84	52.76	61.10	65.40	68.90	45.10	54.04	62.46	66.80	70.46	44.91	53.08	61.72	66.24	69.28
**DNABERT-S**	49.02	56.28	63.85	67.83	70.55	47.85	56.90	64.60	68.52	71.60	48.03	56.11	64.42	68.25	70.98
